# Genetic association and computational analysis of *CYP2R1* gene polymorphisms rs2060793 and rs12794714 with vitamin D deficiency and acute myocardial infarction in the Bangladeshi population: A case control study

**DOI:** 10.1371/journal.pone.0350994

**Published:** 2026-06-05

**Authors:** Sadia Akter, Md. Nazid Bin Ibrahim, Zimam Mahmud, Sonia Tamanna, Md. Shakhawat Hossain Shawon, Farzana Ansari, Md. Zakir Hossain Howlader

**Affiliations:** Laboratory of Nutrition and Health Research, Department of Biochemistry and Molecular Biology, University of Dhaka, Dhaka, Bangladesh; National Institute of Pharmaceutical Education and Research Guwahati, INDIA

## Abstract

Acute myocardial infarction (AMI) remains a leading cause of cardiovascular morbidity and mortality worldwide. Emerging evidence highlights vitamin D as a critical determinant of cardiovascular health. The *CYP2R1* gene encodes the key 25-hydroxylase enzyme responsible for converting vitamin D to its principal circulating metabolite, 25-hydroxyvitamin D. However, the influence of *CYP2R1* polymorphisms on AMI susceptibility, particularly within South Asian populations, has not been well characterized. This study investigates the association of two *CYP2R1* variants, rs2060793 and rs12794714, with AMI risk and their relationship with serum vitamin D levels in a Bangladeshi cohort. A total of 502 participants comprising 251 AMI patients and 251 age- and sex-matched controls were analyzed. Genomic DNA was extracted and genotyped using PCR-RFLP, while serum 25-hydroxyvitamin D_3_ levels were quantified by HPLC. AMI patients exhibited markedly lower vitamin D concentrations (23.92 ± 0.94 ng/mL) than controls (30.3 ± 0.86 ng/mL; *p* < 0.0001). Genotypic analysis revealed a significant association between rs2060793 and AMI risk: the TC (OR = 2.49, 95% CI: 1.34–4.63) and CC (OR = 2.59, 95% CI: 1.37–4.90) genotypes conferred increased susceptibility compared to the TT genotype (*p* = 0.0064). The dominant model (TC + CC vs. TT) further confirmed this relationship (OR = 2.53, 95% CI: 1.39–4.61, *p* = 0.0016). In contrast, rs12794714 showed no significant association with AMI in this population. Stratified analysis indicated that rs2060793 was significantly linked to AMI in males but not females, while both variants were associated with increased risk in individuals aged ≤60 years, but not in those >60 years. Bioinformatic and molecular docking analyses (RegulomeDB, JASPAR, HADDOCK 2.4, DNAproDB) further demonstrated potential regulatory effects of these variants on *CYP2R1* function. Collectively, our findings reveal a novel association between *CYP2R1* rs2060793 and vitamin D deficiency with AMI risk in the Bangladeshi population, underscoring the interplay of genetic and metabolic determinants in the molecular pathogenesis of AMI.

## 1. Introduction

Acute myocardial infarction (AMI), often called a heart attack, happens when blood flow to part of the heart is suddenly blocked. This blockage reduces oxygen supply, leading to damage and death of heart muscle cells. The main cause is usually the rupture or erosion of an atherosclerotic plaque in the coronary arteries, which triggers clot formation and obstructs blood flow [1]. Acute myocardial infarction (AMI) describes the initial stage of heart muscle death caused by ischemia, usually occurring within 1–7 days of symptom onset. It is identified by elevated cardiac biomarkers like troponins, alongside clinical indicators of ischemia such as chest pain or ECG abnormalities [2]. The latest statistics from the World Health Organization (WHO) indicate that cardiovascular diseases account for a large proportion of global mortality, responsible for around 17.9 million deaths annually, which represents 32% of all deaths worldwide. Heart attacks and strokes contribute to 85% of these fatalities [3]. In 2018, the overall prevalence of CVDs in Bangladesh was 5%, with urban areas showing a higher rate (8%) than rural areas (2%) [4]. AMI and stroke were the leading causes of death among hospital inpatients in Bangladesh in 2019 and 2020, highlighting AMI's major role in mortality [5]. Cardiac biomarkers play a crucial role, with troponins serving as the preferred standard. Specifically, Troponin I (cTnI) and Troponin T (cTnT) levels begin to increase 2–6 hours following the onset of an AMI, reach their highest concentration between 24 and 48 hours, and stay elevated for 5–14 days [6].

AMI risk is influenced by variations in genes linked to endothelial function, thrombosis, inflammation, lipid metabolism, and vitamin D metabolism [7–9]. Vitamin D functions as both an endocrine and paracrine signaling molecule, regulating gene expression by downregulating NF-κB and IL-6. This process helps alleviate endothelial inflammation, enhancing flow-mediated dilation and ultimately lowering the risk of atherosclerosis [10]. Vitamin D enhances VEGF expression, which inhibits the initiation of atherosclerotic plaque formation [11] and plays a crucial role in maintaining the physiological equilibrium between blood clot formation (thrombosis) and normal blood coagulation (hemostasis) [12]. Growing evidence indicates that vitamin D plays a protective role in the cardiovascular system by reducing inflammation and modulating the renin–angiotensin–aldosterone system [13]. Calcitriol, the active metabolite of vitamin D, enhances endothelial nitric oxide synthase (eNOS) activity, leading to improved nitric oxide availability and mitigating endothelial dysfunction [14]. Insufficient vitamin D levels contribute to chronic inflammation, which leads to atherosclerosis, plaque instability, and thrombus formation. These are the key mechanisms in developing AMI [10,11]. A deficiency in vitamin D is linked to increased LDL cholesterol, reduced HDL cholesterol, and elevated triglyceride levels, all of which contribute to atherosclerosis and AMI [15]. Additionally, vitamin D is essential for calcium metabolism, preventing vascular calcification and myocardial stiffness. It regulates parathyroid hormone (PTH), which is crucial for cardiomyocyte contractility and vascular function [16].

Plant-based and fortified foods contain Vitamin D2 (ergocalciferol), while vitamin D3 (cholecalciferol) is synthesized in the skin from 7-dehydrocholesterol and obtained from animal-based foods. Both forms are biologically inactive and require two hydroxylation steps for activation. The first occurs in the liver via CYP2R1, forming 25-hydroxyvitamin D (25OHD), and the second in the kidneys via CYP27B1, producing the active hormone calcitriol (1,25-dihydroxyvitamin D). 25OHD circulates bound to vitamin D binding protein (DBP), which also transports calcitriol. Calcitriol functions by regulating gene expression through its interaction with the vitamin D receptor (VDR) in cell nuclei. Inactivation involves further hydroxylation by CYP24A1, generating less active metabolites [17].

The *CYP2R1* gene encodes the enzyme 25-hydroxylase also generally named CYP2R1 (cytochrome P450 2R1) enzyme, which plays a crucial role in the vitamin D metabolic pathway. It is located on chromosome 11p15.2 covering approximately 15.5 kilobases and comprises five exons and four introns [18]. Vitamin D from diet or skin synthesis is inactive and must be converted to 25-hydroxyvitamin D, the main circulating form [17]. The liver is the primary site for this conversion, primarily via the enzyme CYP2R1, which shows significantly higher activity toward vitamin D_3_ compared to other enzymes [19]. While other cytochrome P450 enzymes contribute, CYP2R1 is the dominant 25-hydroxylase in humans [20].

The variant rs12794714, which is a synonymous variant, can cause disruptions in mRNA folding that may result in decreased protein production or misfolded proteins [21]. Additionally, some synonymous variants can not only alter DNA sequences that act as recognition sites for transcription factors, but also can create or eliminate splice sites, leading to abnormal mRNA splicing and the formation of dysfunctional proteins [22,23]. The variant rs2060793, which is an intergenic variant, can also influence disease pathogenesis by affecting gene regulation. These variants may disrupt regulatory elements such as enhancers or silencers, thereby modifying the expression of nearby or even distant genes [24]. Moreover, prior evidence suggests their association with vitamin D levels and cardiovascular risk [25]. The rs2060793 variant has been reported to increase susceptibility to myocardial infarction and cardiovascular diseases in some populations [26,27]. Similarly, rs12794714 has been associated with serum 25(OH)D concentrations and coronary artery disease risk, potentially through modulation of vitamin D metabolism [28]. Minor allele frequency (MAF) data from the 1000 Genomes Project show that rs2060793 has a global MAF of 0.3403 and a South Asian MAF of 0.358, while rs12794714 has a global MAF of 0.3492 and a South Asian MAF of 0.445. The relatively high frequencies of these variants in South Asians support their suitability for association analysis in the Bangladeshi population.

In Bangladesh, the prevalence of acute myocardial infarction (AMI) is rising rapidly and has become a major public health concern. As no prior studies have investigated the potential relationship among vitamin D level, the role of the *CYP2R1* gene and the risk of AMI in the Bangladeshi population, our study aimed to assess the frequency of *CYP2R1* gene polymorphisms in patients with AMI. In addition, we evaluated the impact of these genetic variations on serum 25(OH)D concentrations. The findings from this investigation could contribute to a more personalized approach to AMI prevention and treatment by identifying individuals at higher risk due to impaired vitamin D metabolism, thereby promoting earlier intervention and more effective management strategies.

## 2. Methods

### 2.1 Study subjects

A total of 502 subjects, including 251 patients diagnosed with AMI and 251 healthy individuals, participated in this case-control study. The sample size for the genetic association analysis was determined using Cochran’s formula for estimating proportions in large populations [29]. The exact prevalence of AMI in Bangladesh is not known. One data indicates prevalence of AMI is 3.4% in rural areas and 19.6% in urban areas [30]. According to this, the calculated value (≈246) represents the minimum required sample size per group when equal allocation is used. As the study included 251 cases and 251 controls, the study ensures adequate statistical precision considering probable prevalence. All participants had a Bengali ethnic background. The AMI subjects and control samples were recruited from the Cardiology Department of Ibrahim Cardiac Hospital and Research Institute between 01/06/2023 and 31/12/2023. The diagnosis of AMI was confirmed according to medical reports, including Troponin I levels. The control participants were chosen from the same geographic region, possessing a comparable ethnic background and a similar age range to the AMI group. Patients with chronic liver disease or chronic kidney disease were excluded from the study, as these conditions can significantly affect the result interpretation.

The Ethical Review Committee of the Department of Biochemistry and Molecular Biology, approved the study at the University of Dhaka (BMBDU-ERC/EC/23/09). In addition, we confirm that all methods used in this study were performed following the relevant guidelines and regulations. Informed written consent was taken from all the study subjects before collecting samples.

### 2.2 Sample collection

Blood samples were obtained from both AMI patients (diagnosed based on troponin I levels and ECG findings) and healthy controls under approved protocols of the respective institutions. Diagnosis was based on elevated hs-Troponin I levels (>40 pg/mL) [31,32], demonstrating a rising and/or falling pattern, along with clinical symptoms of myocardial ischemia and characteristic electrocardiographic (ECG) changes. ECG abnormalities included ST-segment abnormality, T-wave inversion, development of pathological Q waves, and new-onset left bundle branch block. In a sterile setting, a trained professional drew venous blood (5 mL) from each subjects using a disposable syringe and transferred it into EDTA-coated tubes. The EDTA blood samples were stored at –20 ℃ until DNA extraction. For serum separation, samples were centrifuged at 3,000 revolutions per minute for 15 minutes using a Digisystem laboratory centrifuge, after which the serum was carefully collected and preserved at –20 ℃ for subsequent analyses.

### 2.3 Vitamin D extraction

For vitamin D analysis, 0.5 mL of serum was transferred into a screw-capped tube, followed by the addition of 350 μL of a methanol:2-propanol mixture (80:20, v/v). The mixture was vortexed for 30 seconds using a Multitude vortex mixer (Digisystem; VH-200) to ensure proper protein precipitation and homogenization. To extract 25-hydroxyvitamin D [25(OH)D], 2 mL of hexane was added, and the extraction procedure was repeated three times, with each cycle involving 60 seconds of vigorous mixing. After extraction, centrifugation (Digisystem) was carried out to promote phase separation. The upper hexane layer containing vitamin D was carefully collected into a conical tube and evaporated to dryness using liquid nitrogen. Using 100 μL of methanol, the residue was dissolved, preparing the sample for subsequent HPLC analysis [33]. As vitamin D is light sensitive, extraction procedures were conducted under controlled laboratory conditions with minimal light exposure. Samples were protected from direct light by using amber-colored tubes.

### 2.4 Determination of 25-Hydroxyvitamin D3 by HPLC

Serum vitamin D levels were determined using HPLC on a Dionex/Thermo UltiMate 3000 system. HPLC peak analysis was performed using the Chromeleon™ Chromatography Data System (CDS) software (Thermo Scientific). Background subtraction and baseline correction were applied to all chromatograms to ensure accurate peak integration and quantification. Separation was performed on a C18 reverse-phase column (250 mm × 4 mm, 5 µm size) maintained at 25 °C, with HPLC-grade methanol as the mobile phase at a flow rate of 1.3 mL/min. A standard vitamin D solution (200 µg/mL; 200 ppm) was prepared in HPLC-grade methanol for calibration. For analysis, 20 µL of each sample was injected, and detection was carried out using a UV detector set at 265 nm. Vitamin D was identified according to its retention time, and quantification was achieved by measuring the corresponding peak area. This method provided reliable and reproducible assessment of serum vitamin D levels across all samples.

### 2.5 DNA extraction

Prior to DNA extraction, the blood samples were gently agitated using a blood mixer (vortex) to ensure uniform mixing. Genomic DNA was then isolated following the previously described protocol [34,35]. The concentration and purity of the extracted DNA were determined by a NanoDrop spectrophotometer.

### 2.6 PCR amplification

PCR amplification was carried out in a total volume of 15 µL, containing 7.5 µL of GoTaq G2 Green Master Mix (Promega Corporation), 5.05 µL of nuclease-free water, 0.45 µL of DMSO, 0.5 µL each of forward and reverse primers, and 1 µL of template DNA. Sequences of primers along with their respective amplicon sizes are listed in S1 Table. Thermal cycling was carried out under conditions previously described [36,37].

### 2.7 Genotyping

The enzymes Hinfl and Ddel was chosen for PCR-RFLP investigations of rs2060793 and rs12794714 respectively utilising NEBcutter V3.0, an internet-based tool for SNP-RFLP analysis [38]. The 423 base pair and 448 bp PCR product respectively underwent restriction digestion under the optimal reaction conditions specified by the manufacturer's methods. The process of restriction enzyme digestion was carried out according to the established digestion procedure [39,40]. The PCR products that specifically target rs2060793 and rs12794714 were subjected to a 16-hour digestion process using the Hinfl and Ddel enzyme respectively at a temperature of 37°C.

For the rs2060793 genetic variant, individuals with the TT genotype show a single fragment of 365 base pairs. Those with the TC genotypes display fragments of 365 bp,263 bp, 102 bp, and 58 bp. Those with the TC genotypes display fragments of 263 bp, 102 bp, and 58 bp. For the rs12794714 variant, the CC genotype yield a 448 bp fragment. CT genotypes yield fragments of 448 bp, 321 bp and 127 bp. TT genotypes yield fragments of 321 bp and 127 bp. The PCR products digested by HinfI were subjected to electrophoresis on a 4% agarose gel at a voltage of 65 volts for a duration of 60 minutes. The PCR products digested by Ddel were akin resolved on a 3% agarose gel at a voltage of 70 volts for a duration of 50 minutes. Gel Documentation System used UV light to examine the stained digested products after they had been soaked in EtBr solution for 15–20 minutes.

To ensure quality of genotyping, negative controls were included in each PCR run to detect contamination. In addition, a subset of samples was re-genotyped blindly, yielding 100% reproducibility. Approximately 5% of randomly selected samples representing all genotype categories were validated by Sanger sequencing.

### 2.8 Sequencing of PCR products

To ensure the accuracy of PCR-RFLP genotyping, 5% of randomly selected PCR products from both case and control samples underwent Sanger sequencing with the Barcode-tagged Sequencing (BTSeq) method. Chromatogram data were analyzed using Geneious Prime 2022.2 software, based on previously established procedures [41,42].

### 2.9 Statistical analysis

SNPStats online server (https://www.snpstats.net/), software GraphPad Prism version-8.0 and RStudio was used to assess and represent the observed results in a statistically resolved manner. All data including demographic, clinicopathological, vitamin D levels were given as Means ± SEM. Odds ratios (OR), as a measure of relative risk, at 95% confidence intervals (95% CI) were estimated using these tools. GraphPad Prism version 8.0 was also used to perform unpaired T test and Chi-square test to analyze the difference in means between continuous variables and association between different categorial variables respectively. Also, p-value less than 0.05 has been considered statistically significant.

### 2.10 In silico analysis

RegulomeDB (https://www.regulomedb.org/regulome-search/) was employed to evaluate the functional relevance of the variants [43,44]. JASPAR (https://jaspar.elixir.no/) was used to analyze transcription factor binding motifs, allowing the prediction of whether the studied variants could disrupt or create binding sites, thereby influencing gene regulation [45]. The interacting transcription factor (protein) and the corresponding DNA sequence, including the region containing rs2060793, were predicted as a binding site using JASPAR. The DNA structures (wild-type and rs2060793 mutant) were modeled using UCSF Chimera [46]. The interacting protein structure which was HNF4α (PDB ID: 4IQR) was prepared using BIOVIA Discovery Studio 2024. This preparation included cleaning the structure by removing water molecules, adding missing hydrogens, and optimizing the protein’s geometry through energy minimization to ensure a stable conformation for docking [47]. The DNA-binding domain of HNF4α (amino acids 57–132) was identified using UniProt annotations [48]. Docking of HNF4α with the wild-type and rs2060793 mutant DNA was performed using HADDOCK 2.4 (https://rascar.science.uu.nl/haddock2.4/) [49,50]. The resulting docked complexes were analyzed using DNAproDB (https://dnaprodb.usc.edu/) to characterize the protein-DNA interfaces [51–53].

## 3. Results

### 3.1 Demographic and clinical data of study subjects

A total of 251 control subjects (180 males, 71 females) were enrolled in the study. Similarly, a total of 251 AMI patients (180 males, 71 females) were enrolled in the study. Demographic data of all subjects were obtained using a standardized questionnaire. Age distributions were similar, with a mean age of 59.32 ± 0.54 years in controls and 60.1 ± 0.47 years in cases (p = 0.3297, not significant). The case group exhibits markedly elevated hsTroponin I (3918 ± 471 pg/mL vs. 1.845 ± 0.079 pg/mL in controls, p < 0.0001), indicating severe cardiac injury. Lipid profiles further distinguish the groups with higher LDL (99.18 ± 3.29 mg/dL vs. 73.58 ± 1.31 mg/dL, p < 0.0001), elevated triglycerides (178.2 ± 8.34 mg/dL vs. 127.6 ± 1.15 mg/dL, p < 0.0001), and increased Total Cholesterol (172.9 ± 4.09 mg/dL vs. 147.9 ± 1.34 mg/dL, p = 0.0004. On the other hand, cases showing lower HDL (37.63 ± 0.81 mg/dL) than controls (38.7 ± 0.16 mg/dL, p = 0.0066). The demographic and clinical data have been presented in Table 1.

**Table 1:** Demographic and clinicopathological features of study subjects.

Demographic and Clinical Data			
Parameters	Control (n = 251)	Case (n = 251)	p-value
**Age (Year)**	59.32 ± 0.54	60.1 ± 0.47	0.3297(ns)
**Age Group,**			
**<60**	48.64 ± 1.26	50.06 ± 0.97	0.2704(ns)
**>60**	65.17 ± 0.38	65.96 ± 0.61	0.2301 (ns)
**Gender, n (%)**			>0.9999(ns)
**Male**	180 (71.71%)	180 (71.71%)	
**Female**	71 (28.29%)	71 (28.29%)	
**hsTroponin I (pg/mL)**	1.845 ± 0.079	3918 ± 471	<0.0001
**Lipid Profile (mg/dL)**			
**HDL**	38.7 ± 0.16	37.63 ± 0.81	0.0066
**LDL**	73.58 ± 1.31	99.18 ± 3.29	<0.0001
**TG**	127.6 ± 1.15	178.2 ± 8.34	<0.0001
**Total Cholesterol**	147.9 ± 1.34	172.9 ± 4.09	0.0004

p < 0.05 was considered as level of significance. Result is represented as Mean± SEM

### 3.2 Vitamin D Status in Patient and Control Subjects

**Fig 1:**
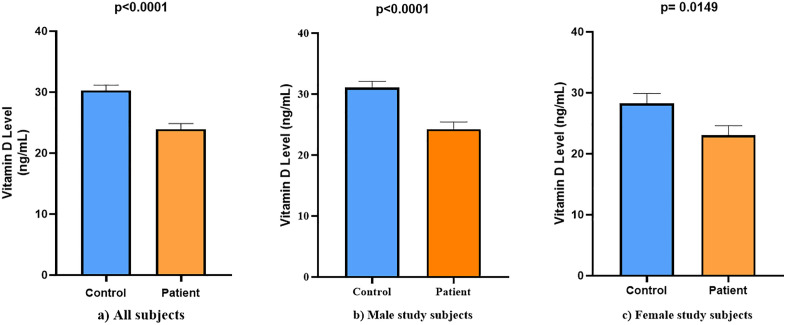
Variation in vitamin D levels between patient and control groups. a) All subjects, b) Male subjects, c) Female subjects. Values are expressed as Mean ± SEM. A p-value of <0.05 was considered statistically significant.

The average vitamin D level among controls was 30.3 ng/mL, with a standard error of 0.8579 ng/mL. In contrast, the mean vitamin D level in AMI patients was notably lower at 23.92 ng/mL, with a standard error of mean of 0.9389 ng/mL. This substantial difference was found to be statistically significant, as indicated by a p-value of p < 0.0001. In males, mean vitamin D levels were significantly lower in patients (24.27 ± 1.16 ng/mL) than in controls (31.09 ± 1.01 ng/mL; p = 0.0001). Similarly, in females, patients had lower levels (23.05 ± 1.56 ng/mL) compared to controls (28.28 ± 1.63 ng/mL; p = 0.0149). Differences in Vitamin D levels between control and case study subjects of different groups is depicted in Fig 1.

**Fig 2:**
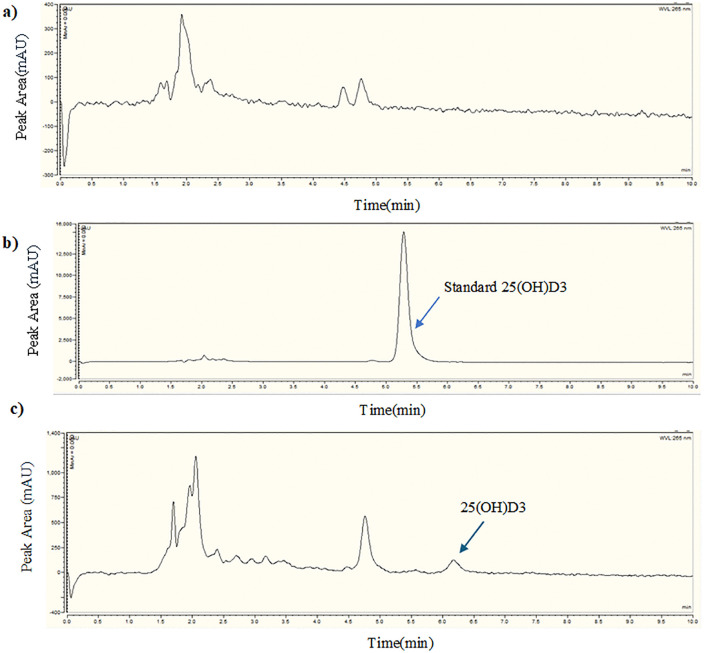
Identification of vitamin D peak in chromatogram. (a) Blank methanol solvent chromatogram, (b) Vitamin D standard solution chromatogram, (c) Sample vitamin D solution chromatogram.

These findings suggest a potential association between lower vitamin D levels and the occurrence of AMI. Chromatogram peaks of blank, standard and sample vitamin D solution are given in Fig 2.

### 3.3 Association between serum vitamin D levels and lipid profile

**Fig 3:**
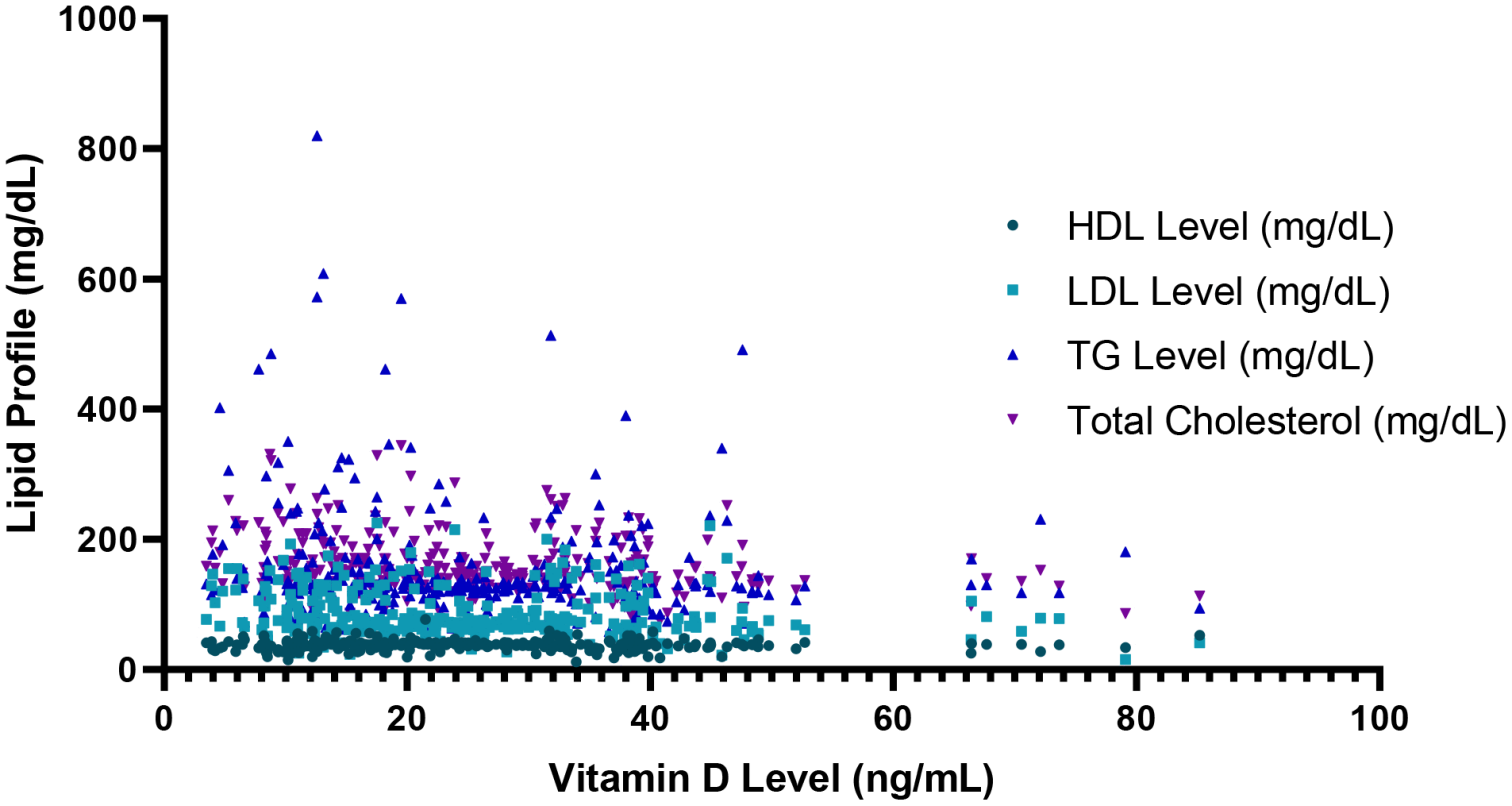
Correlation between serum vitamin D levels and lipid parameters. This scatter plot shows the relationship between serum Vitamin D levels (ng/mL) on the x-axis and different lipid profile parameters (mg/dL) on the y-axis, including HDL, LDL, triglycerides (TG), and total cholesterol.

There is a significant negative correlation between vitamin D levels and LDL cholesterol (r = –0.175, p = 0.001), triglycerides (r = –0.151, p = 0.004), and total cholesterol (r = –0.225, p < 0.0001). However, no significant association was observed with HDL cholesterol (r = –0.034, p = 0.275). These findings suggest association between lower vitamin D levels and adverse lipid profiles Fig 3.

### 3.4 Frequency distribution in different categories of vitamin D status

**Fig 4:**
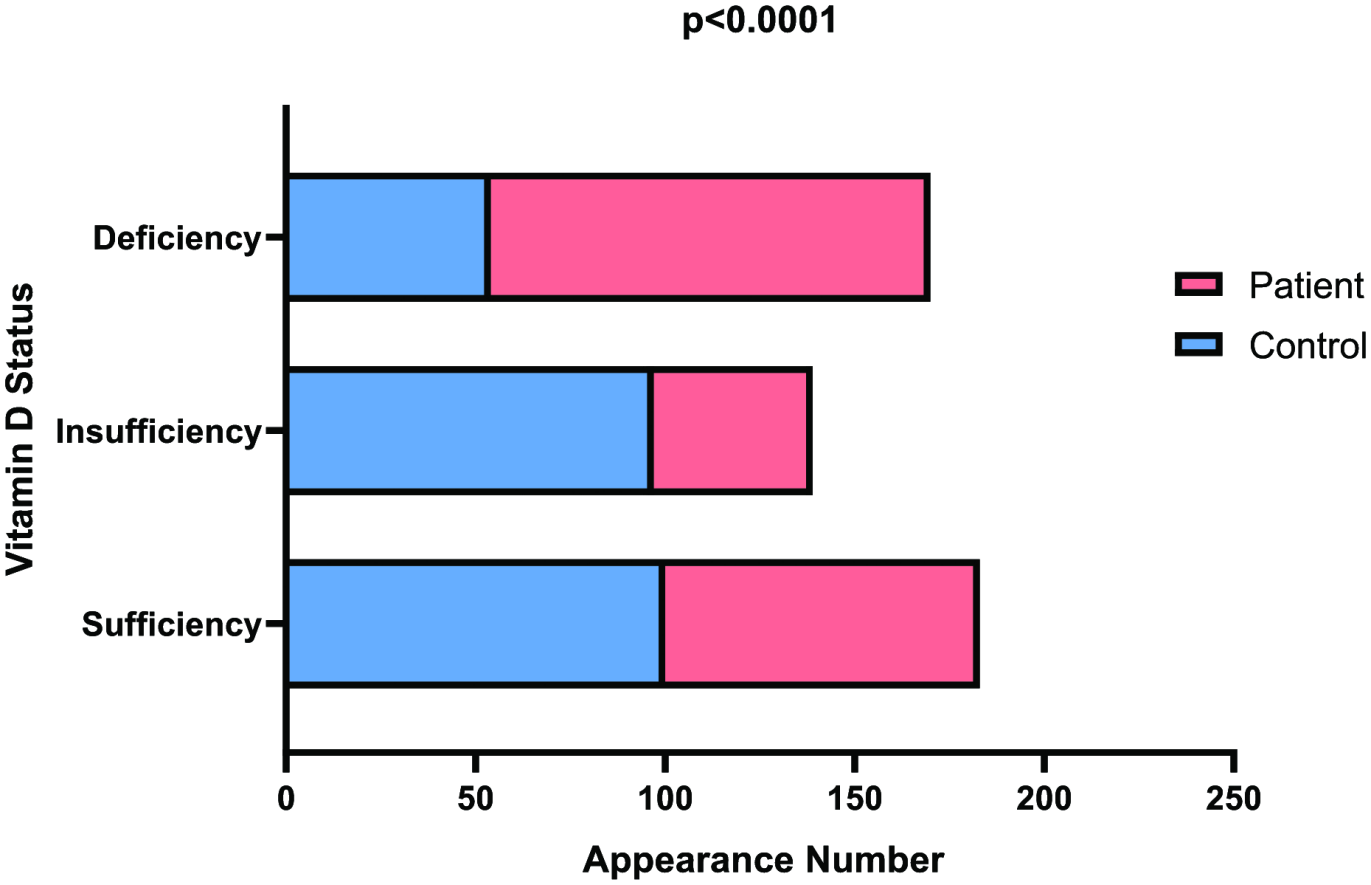
Frequency distribution in different categories of vitamin D status. Chi-square was performed to analyze data. p < 0.05 was considered as level of significance. Significant differences were observed in every category of vitamin D status indicating patients having grater deficiencies in vitamin D than control subjects (p < 0.0001).

The distribution of vitamin D levels between patients and control subjects was examined to assess sufficiency (≥30 ng/mL), insufficiency (20–29 ng/mL), and deficiency (<20 ng/mL) according to established clinical guidelines [54]. For those deemed ‘Sufficient’ in vitamin D levels, 39.8% of control subjects and 34.4% of fell into this category, while 38.6% of control and 17.4% of patients were categorized as having insufficient vitamin D levels. On the contrary, 21.5% of control and 48.1% of patients belong to the “deficient” category. The chi-square test was applied to assess the association between vitamin D status (sufficient, insufficient, and deficient) and patient status (control or patient). p < 0.05 was considered as level of significance. The analysis revealed a statistically significant association, with a p-value<0.0001 Fig 4.

### 3.5 Assessment of relationship between rs2060793 and AMI risk

**Fig 5:**
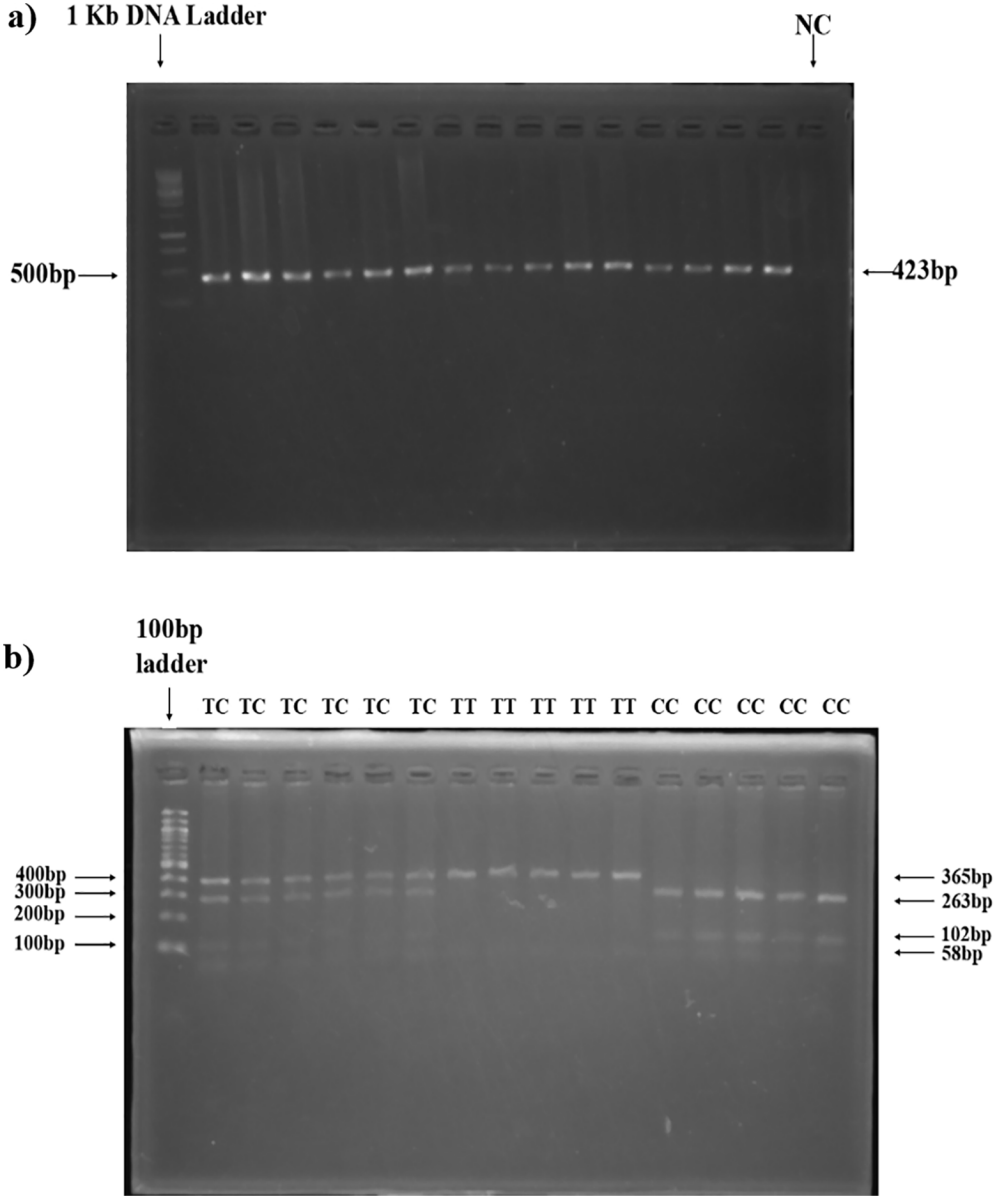
Visualization of PCR and restriction digested products (rs2060793). a) A 423 bp amplicon of the *CYP2R1* gene, targeting the rs2060793 polymorphism, b) Restriction digestion products of TT (365 bp, 58 bp), TC (365 bp, 263 bp, 102 bp and 58 bp) and CC (263 bp, 102 bp and 58 bp) genotypes.

In the genotypic frequency distribution of rs2060793, 15.5%, 50.2% and 34.3% control subjects were respectively homozygous wild (TT), heterozygous mutant (TC) and homozygous variant (CC). Among the case subjects, the occurrences were 6.8% for homozygous native (TT), 54.6% for heterozygous variant (TC) and 38.6% for homozygous variant (CC) (Table 2). Highly significant difference (p-value = 0.0076) found among the study subjects’ genotypic arrangement by performing Chi-square test. PCR products and restriction digestion products of different genotypes of rs2060793 on agarose gel are shown in Fig 5(a) and 5(b).

**Table 2:** Frequency distribution of rs2060793 in AMI patients and controls.

Genetic Model	Genotypes	Controls (n = 251) n(%)	Patients (n = 251) n(%)	p-value	Odds Ratio	95% Confidence Interval
**Co-dominant Model**	TT	39 (15.5%)	17 (6.8%)	0.0076	1(Ref.)	
	TC	126 (50.2%)	137(54.6%)		2.49	1.34 to 4.63
	CC	86 (34.3%)	97 (38.6%)		2.59	1.37 to 4.90
**Dominant Model**	TT	39 (15.5%)	17 (6.8%)	0.0016	1(Ref.)	
	TC + CC	212 (84.5%)	234(93.2%)		2.53	1.39 to 4.61
**Recessive Model**	TT + TC	165 (65.7%)	154(61.4%)	0.31	1(Ref.)	
	CC	86 (34.3%)	97 (38.6%)		1.21	0.84 to 1.74

p < 0.05 was considered as level of significance. Result is represented as Mean± SEM

In the examination of the *CYP2R1* rs2060793 genetic model and genotypic frequencies for both AMI patients and control subjects, several key findings emerged. The co-dominant model shows that individuals with the TC genotype have 2.49 times higher odds of AMI compared to the TT genotype (Wild-type) group, with a confidence interval ranging from 1.34 to 4.63. Similarly, individuals with the CC genotype have 2.59 times higher odds of AMI compared to the TT genotype, with a confidence interval of 1.37 to 4.90. The observed association is statistically significant (p = 0.0064). In the Dominant Model, the TT genotype was present in 15.5% of controls and 6.8% of AMI patients. The TC + CC genotype combination was more frequent, comprising 84.5% of controls and 93.2% of AMI patients, with a p-value 0.0016 and an OR of 2.53, along with a 95% CI spanning from 1.39 to 4.61. In the Recessive Model, the TT + TC genotype group constituted 65.7%% of controls and 61.4% of AMI patients. The CC genotype was observed in 34.3% of controls and 38.6% of AMI patients. This difference is also not statistically significant (p = 0.31), with an OR of 1.21 and a 95% CI ranging from 0.84 to 1.74. Table 2 presents the genotype distributions of the two SNPs among the study subjects.

### 3.6 Assessment of relationship between rs12794714 and AMI Risk

**Fig 6:**
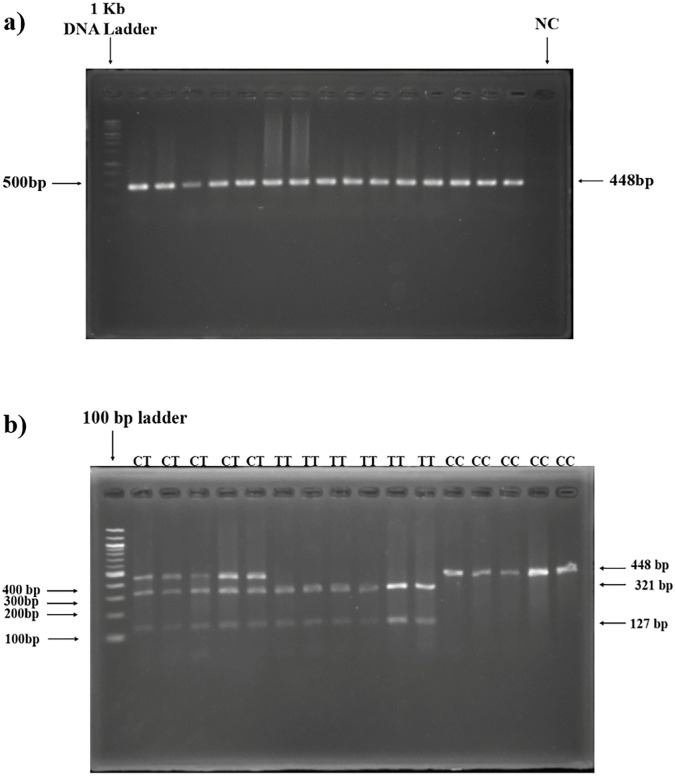
Visualization of PCR and restriction digested products (rs12794714). a) PCR product of the desired 448 bp *CYP2R1* gene segment amplicon targeting rs12794714, b) Restriction digestion products of CC (448 bp), CT (448 bp, 321 bp and 127 bp) and TT (321 bp and 127 bp) genotypes.

In the genotypic frequency distribution of rs12794714, 30.7%, 49% and 20.3% control subjects were respectively homozygous wild (CC), heterozygous mutant (CT) and homozygous variant (TT). Among the case subjects, the frequencies were 24.5% for CC, 52.6% for CT and 22.9% for TT. No significant difference (p-value = 0.2964) found among the study subjects’ genotypic arrangement by performing Chi-square test. PCR products and restriction digestion products of different genotypes of rs2060793 on agarose gel are shown in Fig 6(a) and 6(b).

In the examination of the *CYP2R1* rs12794714 genetic models for both AMI patients and control subjects, there is no significant differences in any model which is depicted in Table 3.

**Table 3:** Frequency distribution of rs12794714 genotypes in AMI patients and control subjects.

Genetic Model	Genotype Status	Control (n = 251) n (%)	Case (n = 251) n (%)	p-value	Odds Ratio	95% CI
**Co-dominant Model**	CC	77 (30.7%)	61 (24.3%)	0.30	1(Ref.)	
	CT	123 (49%)	133 (53%)		1.36	0.90 to 2.07
	TT	51 (20.3%)	57 (22.7%)		1.41	0.85 to 2.34
**Dominant Model**	CC	77 (30.7%)	61 (24.3%)	0.11	1(Ref.)	
	CT + TT	174 (69.3%)	190(75.7%)		1.38	0.93 to 2.04
**Recessive Model**	CC + CT	200 (79.7%)	194(77.3%)	0.51	1((Ref.)	
	TT	51 (20.3%)	57 (22.7%)		1.15	0.75 to 1.76

p < 0.05 was considered as level of significance. Result is represented as Mean± SEM

### 3.7 Allele frequency distribution of both SNPs of *CYP2R1*

The analysis of allele frequencies revealed notable differences between control and AMI case groups. For rs2060793, the C allele was more frequent than the T allele in both groups, accounting for 59% in controls and increasing to 66% in AMI patients, while the T allele frequencies were 41% and 34%, respectively. This difference in allele distribution was statistically significant (p = 0.0313), indicating a potential association with AMI risk.

**Fig 7:**
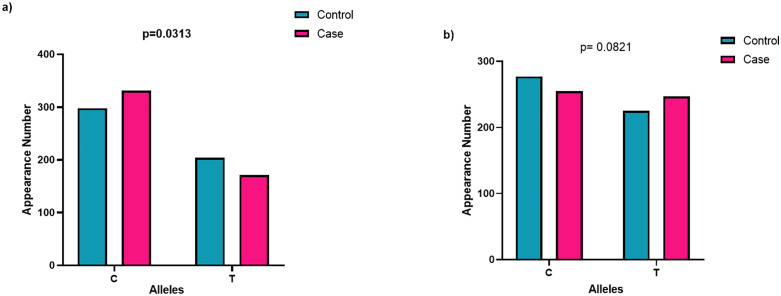
a) Allelic Frequency distribution of rs2060793 and b) rs12794714 in study subjects.

In contrast, for rs12794714, the C allele was slightly more frequent in controls (55%) compared to AMI cases (51%), while the T allele was present at 45% and 49%, respectively. However, this variation in allele frequencies did not reach statistical significance (p = 0.0821), suggesting no strong association with disease status in the studied population. The distributions of alleles in both single nucleotide polymorphisms in controls and patients are shown in Fig 7.

### 3.8 Association between AMI and both SNPs in different age groups

Among participants aged 60 years or younger, both rs2060793 and rs12794714 demonstrated significant associations with AMI under co-dominant and dominant genetic models. For rs2060793, individuals with the TC genotype had an OR of 4.72 (95% CI: 1.71–13.04), and those with the CC genotype had an OR of 4.35 (95% CI: 1.56–12.16) compared to TT (p = 0.003). The dominant model (TC + CC vs TT) showed an OR of 4.55 (95% CI: 1.69–12.26; p = 0.0007).

For rs12794714, the CT genotype had an OR of 1.88 (95% CI: 1.07–3.32) and TT had an OR of 2.11 (95% CI: 1.04–4.29) compared to CC (p = 0.048). The dominant model (CT + TT vs CC) also showed a significant association with an OR of 1.94 (95% CI: 1.13–3.34; p = 0.015).

No significant associations were found in the > 60 years age group for either SNP under any genetic model. A comparative overview is given on Table 4 (a) and Table 4 (b).

**Table 4:** (a): Frequency distribution of *CYP2R1* rs2060793 and rs12794714 genotypes in study subjects aged 60 or below. (b): Frequency distribution of *CYP2R1* rs2060793 and rs12794714 genotypes in study subjects aged over 60.

SNPs	Genetic Model	Genotypes	Controls (n = 158) n (%)	Patients (n = 126) n (%)	p-value	Odds Ratio	95% CI
**rs2060793**	**Co-dominant Model**	TT	25 (15.8%)	5 (4%)	0.003	1(Ref.)	
		TC	71 (44.9%)	67 (53.2%)		4.72	1.71 to 13.04
		CC	62 (39.2%)	54 (42.9%)		4.35	1.56 to 12.16
	**Dominant Model**	TT	25 (15.8%)	5 (4%)	7.00E-04	1(Ref.)	
		TC + CC	133 (84.2%)	121 (96%)		4.55	1.69 to 12.26
	**Recessive Model**	TT + TC	96 (60.8%)	72 (57.1%)	0.54	1((Ref.)	
		CC	62 (39.2%)	54 (42.9%)		1.16	0.72 to 1.87
**rs12794714**	**Co-dominant Model**	CC	53 (33.5%)	26 (20.6%)	0.048	1(Ref.)	
		CT	78 (49.4%)	72 (57.1%)		1.88	1.07 to 3.32
		TT	27 (17.1%)	28 (22.2%)		2.11	1.04 to 4.29
	**Dominant Model**	CC	53 (33.5%)	26 (20.6%)	0.015	1(Ref.)	
		CT + TT	105 (66.5%)	100 (79.4%)		1.94	1.13 to 3.34
	**Recessive Model**	CC + CT	200 (79.7%)	194(77.3%)	0.28	1((Ref.)	
		TT	51 (20.3%)	57 (22.7%)		1.39	0.77 to 2.5
**(b): Frequency distribution of *CYP2R1* rs2060793 and rs12794714 genotypes in study subjects aged over 60.**							
**SNPs**	**Genetic Model**	**Genotypes**	**Controls** **(n = 93)** **n (%)**	**Patients** **(n = 125)** **n (%)**	**p-value**	**Odds** **Ratio**	**95% CI**
**rs2060793**	**Co-dominant Model**	TT	14 (15.1%)	12 (9.6%)	0.26	1(Ref.)	
		TC	55 (59.1%)	70 (56%)		1.48	0.64 to 3.47
		CC	24 (25.8%)	43 (34.4%)		2.09	0.83 to 5.24
	**Dominant Model**	TT	14 (15.1%)	12 (9.6%)	0.22	1(Ref.)	
		TC + CC	79 (85%)	113(90.4%)		1.67	0.73 to 3.80
	**Recessive Model**	TT + TC	69 (74.2%)	82 (65.6%)	0.17	1((Ref.)	
		CC	24 (25.8%)	43 (34.4%)		1.51	0.83 to 2.73
**rs12794714**	**Co-dominant Model**	CC	24 (25.8%)	35 (28%)	0.88	1(Ref.)	
		CT	45 (48.4%)	61 (48.8%)		0.93	0.49 to 1.77
		TT	24 (25.8%)	29 (23.2%)		0.83	0.39 to 1.75
	**Dominant Model**	CC	24 (25.8%)	35 (28%)	0.72	1(Ref.)	
		CT + TT	69 (74.2%)	90 (72%)		0.89	0.49 to 1.64
	**Recessive Model**	CC + CT	69 (74.2%)	96 (76.8%)	0.66	1((Ref.)	
		TT	24 (25.8%)	29 (23.2%)		0.87	0.47 to 1.62

p < 0.05 was considered as level of significance. Result is represented as Mean± SEM.

### 3.9 Association between AMI and both SNPs in different gender groups

The association of *CYP2R1* rs2060793 with AMI was assessed using co-dominant, dominant, and recessive models in both male and female study populations. In males, the co-dominant model revealed a strong and statistically significant association: individuals with the TC genotype had 3.33 times higher odds (95% CI: 1.49–7.45) and those with the CC genotype had 3.39 times higher odds (95% CI: 1.48–7.75) of developing AMI while TT genotype is the reference (p = 0.0064). The dominant model also showed a significant association, with an OR of 3.35 (95% CI: 1.53–7.35; *p* = 0.0012) when comparing the combined TC + CC genotypes to TT. However, the recessive model did not demonstrate statistical significance (OR = 1.21; 95% CI: 0.79–1.86; *p* = 0.38). On the other hand, in females, no statistically significant associations were observed across any of the models. A comparative overview is given on Table 5.

**Table 5:** Frequency distribution of *CYP2R1* rs2060793 genotypes in male and female subjects.

Subjects	Genetic Model	Genotypes	Controls n (%)	Patients n (%)	p-value	Odds Ratio	95% CI
**Male** **(n = 180)**	**Co-dominant Model**	TT	27 (15%)	9 (5%)	0.0064	1(Ref.)	
		TC	91 (50.6%)	101 (56.1%)		3.33	1.49 to 7.45
		CC	62 (34.4%)	70 (38.9%)		3.39	1.48 to 7.75
	**Dominant Model**	TT	27 (15%)	9 (5%)	0.0012	1(Ref.)	
		TC + CC	153 (85%)	171 (95%)		3.35	1.53 to 7.35
	**Recessive Model**	TT + TC	118 (65.6%)	110 (61.1%)	0.38	1(Ref.)	
		CC	62 (34.4%)	70 (38.9%)		1.21	0.79 to 1.86
**Female** **(n = 71)**	**Co-dominant Model**	CC	26 (36.6%)	19 (26.8%)	0.4	1(Ref.)	
		CT	33 (46.5%)	36 (50.7%)		1.49	0.70 to 3.18
		TT	12 (16.9%)	16 (22.5%)		1.82	0.7 to 4.74
	**Dominant Model**	CC	26 (36.6%)	19 (26.8%)	0.21	1(Ref.)	
		CT + TT	45 (63.4%)	52 (73.2%)		1.58	0.77 to 3.23
	**Recessive Model**	CC + CT	59 (83.1%)	55 (77.5%)	0.4	1((Ref.)	
		TT	12 (16.9%)	16 (22.5%)		1.43	0.62 to 3.29

p < 0.05 was considered as level of significance. Result is represented as Mean± SEM

The association of *CYP2R1* rs12794714 genotypes with AMI risk was analyzed using co-dominant, dominant, and recessive genetic models. In males, the co-dominant model yielded an OR of 1.31 (CT vs. CC) and 1.28 (TT vs. CC), with both results not statistically significant (p = 0.57). The dominant model (CT + TT vs. CC) showed an OR of 1.30 (95% CI: 0.81–2.09; p = 0.28), while the recessive model (TT vs. CC + CT) produced an OR of 1.07 (95% Confidence Interval: 0.65–1.75; p = 0.80). On the other hand, in female individuals, the dominant model showed an OR of 1.58 (95% CI: 0.77–3.23; p = 0.21), and the recessive model had an odds ratio of 1.43 (95% CI: 0.62–3.29; p = 0.40). Although all models suggested a trend toward increased AMI risk in variant genotypes, none of the associations reached statistical significance. A comparative overview is given in S2 Table.

### 3.10 Age- and gender-adjusted association between SNPs and disease risk

After adjustment for age and gender, rs2060793 remained significantly associated with disease risk. In the co-dominant model, both TC (OR = 2.71, 95% CI: 1.42–5.17) and CC (OR = 3.19, 95% CI: 1.63–6.24) genotypes showed significantly increased risk compared to TT (p = 0.0018). The dominant model (TC + CC vs TT) also showed a significant association (OR = 2.89, 95% CI: 1.55–5.40; p = 5.00E-04), while the recessive model was not significant (p = 0.094). On the other hand, rs12794714 showed no significant association in any genetic model after adjustment (all p > 0.05). These findings indicate that rs2060793 is independently associated with disease risk even after controlling for age and gender, whereas rs12794714 is not significantly associated. The details are given in the following Table 6. After performing model diagnostic assessments using RStudio, it was found that Variance Inflation Factor (VIF) analysis showed no evidence of significant multicollinearity among predictor variables in either regression model, with all adjusted GVIF values close to 1. The Hosmer–Lemeshow goodness-of-fit test indicated acceptable fit for the rs2060793 model (p = 0.6175). In contrast, the rs12794714 model showed a significant lack of fit (p = 0.0084), suggesting that the model may not fully capture the observed data distribution. Notably, rs12794714 also did not show a significant association with MI risk in the adjusted logistic regression analysis.

**Table 6:** Frequency distribution of rs2060793 and rs12794714 after adjustment for age and gender.

SNPs	Genetic Model	Genotypes	Odds Ratio	95% CI	p-value
**rs2060793**	**Co-dominant Model**	TT	1(Ref.)		0.0018
		TC	2.71	1.42 to 5.17	
		CC	3.19	1.63 to 6.24	
	**Dominant Model**	TT	1(Ref.)		5.00E-04
			TC + CC	2.89	1.55 to 5.40
	**Recessive Model**	TT + TC	1((Ref.)		0.094
			CC	1.39	0.94 to 2.06
**rs12794714**	**Co-dominant Model**	CC	1(Ref.)		0.54
		CT	1.28	0.83 to 1.99	
		TT	1.19	0.7 to 2.04	
	**Dominant Model**	CC	1(Ref.)		0.28
			CT + TT	1.26	0.83 to 1.9
	**Recessive Model**	CC + CT	1((Ref.)		0.95
			TT	1.02	0.65 to 1.6

p < 0.05 was considered as level of significance.

### 3.11 Effect of genotypes on lipid parameters in the study subjects

The association between genotypes and lipid parameters was evaluated using the Kruskal–Wallis test. This non-parametric test was applied because lipid parameters did not follow a normal distribution and comparisons were required across three independent genotype groups.

For rs2060793, the mean HDL level ± SEM values were 38.27 ± 1.20 mg/dL (TT), 38.55 ± 0.77 mg/dL (TC), and 37.37 ± 0.68 mg/dL (CC). Although the CT genotype showed slightly higher HDL levels and CC showed marginally lower levels, the difference was not statistically significant (Kruskal–Wallis, p = 0.6759). LDL levels demonstrated a noticeable trend, with mean ± SEM values of 80.75 ± 4.08 mg/dL (TT), 93.06 ± 3.14 mg/dL (TC), and 85.32 ± 3.52 mg/dL (CC). The TC genotype exhibited comparatively higher LDL levels than TT and CC genotypes. However, this trend did not reach statistical significance (p = 0.0819), though it approached borderline significance. Triglyceride levels showed mean ± SEM values of 149.0 ± 14.24 mg/dL (TT), 152.7 ± 6.39 mg/dL (TC), and 166.5 ± 9.60 mg/dL (CC). A gradual increasing trend was observed from TT to CC genotype; however, the difference was not statistically significant (p = 0.3221). Similarly, total cholesterol levels were 156.5 ± 5.82 mg/dL (TT), 164.1 ± 3.82 mg/dL (TC), and 162.8 ± 4.13 mg/dL (CC). Although TC carriers exhibited slightly higher total cholesterol levels, no significant difference was detected among the genotypes (p = 0.7332).

In the case of rs12794714, for HDL levels, the mean ± SEM values were 37.89 ± 0.94 mg/dL (CC), 38.60 ± 0.71 mg/dL (CT), and 36.82 ± 0.86 mg/dL (TT). No statistically significant difference was observed among the genotypes (Kruskal–Wallis, p = 0.5559). Similarly, LDL levels showed mean ± SEM values of 87.22 ± 3.72 mg/dL (CC), 88.94 ± 2.96 mg/dL (CT), and 90.04 ± 5.28 mg/dL (TT), even though there is an increasing trend but the variation between groups is not significant (p = 0.8701). Triglyceride levels were 161.3 ± 11.42 mg/dL (CC), 158.0 ± 6.64 mg/dL (CT), and 150.5 ± 10.57 mg/dL (TT), and the difference was not statistically significant (p = 0.6433). Total cholesterol levels were 162.3 ± 4.74 mg/dL (CC), 163.9 ± 3.57 mg/dL (CT), and 159.8 ± 5.94 mg/dL (TT), with no significant difference among the genotypes (p = 0.6273).

**Fig 8:**
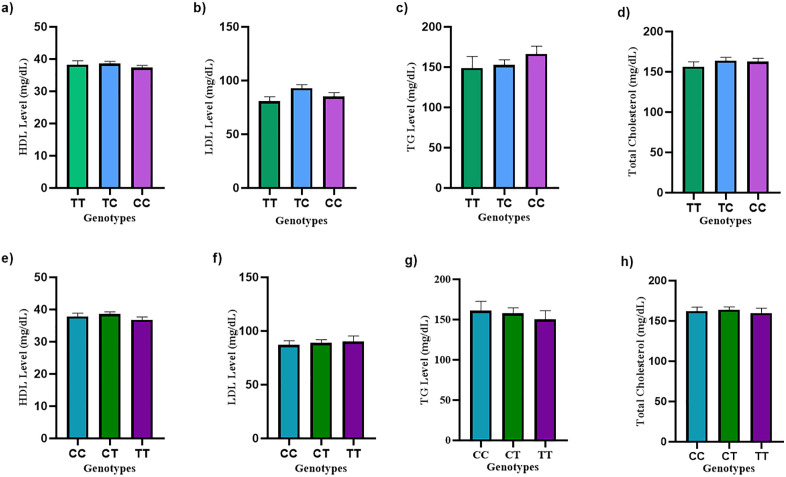
Lipid parameters across different genotypes. (a–d) Lipid parameters across rs2060793 genotypes: (a) HDL, (b) LDL, (c) Triglycerides (TG), and (d) Total cholesterol. (e–h) Lipid parameters across rs12794714 genotypes: (e) HDL, (f) LDL, (g) Triglycerides (TG), and (h) Total cholesterol. Values are presented as mean ± SEM; statistical comparisons were performed using the Kruskal–Wallis test.

Overall, neither rs2060793 nor rs12794714 genotypes demonstrated a statistically significant association with HDL, LDL, triglyceride, or total cholesterol levels in the study population, although modest genotype-specific trends were observed for LDL and TG levels. The relationship between genotypes and lipid parameters is depicted in Fig 8a-8h).

### 3.12 Effect of genotypes on Vitamin D level in the study subjects

**Fig 9:**
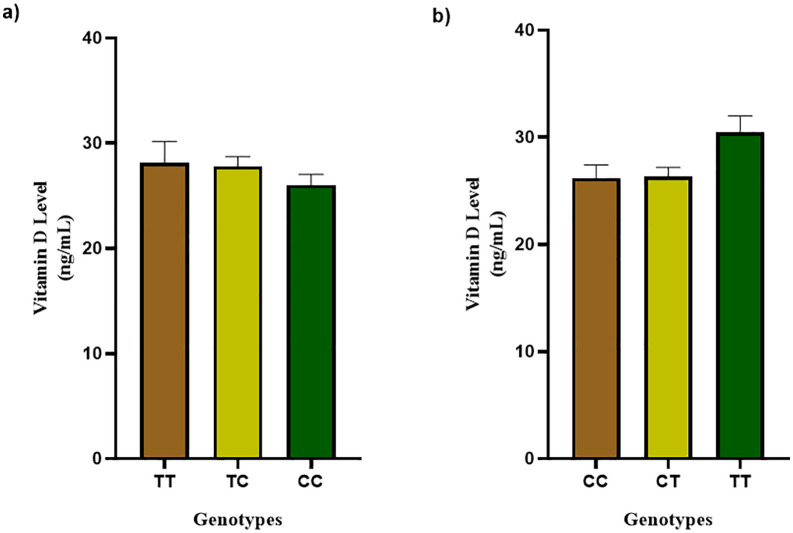
Vitamin D levels across different genotypes. (a) rs2060793, (b) rs12794714. Values are presented as mean ± SEM; statistical comparisons were performed using the Kruskal–Wallis test.

For rs2060793, no statistically significant difference was observed among genotypes (TT: 28.10 ± 2.046, TC: 27.79 ± 0.9223, CC: 26.02 ± 1.017; P = 0.4173). Although the difference in circulating vitamin D levels across rs2060793 genotypes did not reach statistical significance, a decreasing trend was observed. Notably, individuals carrying the homozygous mutant genotype exhibited comparatively lower vitamin D levels. In contrast, rs12794714 mutant genotypes demonstrated higher vitamin D levels and the difference between genotype groups was significant (CC: 26.19 ± 1.220, CT: 26.32 ± 0.8646, TT: 30.44 ± 1.545; P = 0.0392). A comparative overview is given in Fig 9. Individual level genotype and vitamin D data is given in S3 Table.

### 3.13 Validation of PCR-RFLP genotyping by sequencing

**Fig 10:**
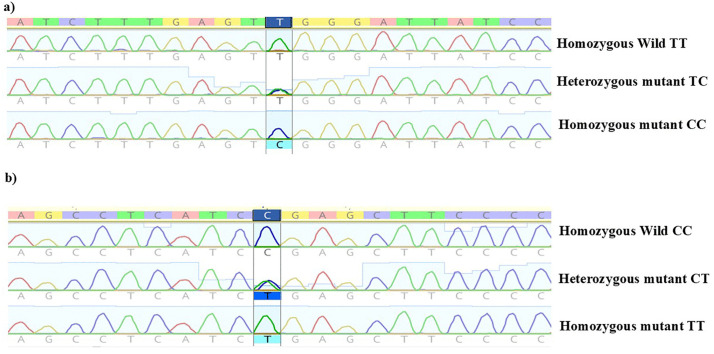
Chromatogram peaks from sequencing outcomes of different genotypes of a) rs2060793 and b) rs12794714.

PCR products representing different genotypes of both SNPs were analyzed using Sanger sequencing, and the results were fully consistent with the PCR-RFLP findings. Representative chromatograms are presented in Fig 10(a) and 10(b).

### 3.14 Hardy-Weinberg equilibrium analysis

The Hardy-Weinberg equilibrium (HWE) analysis revealed that for rs2060793, the overall and case groups significantly deviated from HWE (p < 0.05), suggesting a potential association with AMI, while the control group remained in equilibrium. In contrast, rs12794714 showed no deviation from HWE in the overall, control, or case groups (p > 0.05), indicating stable genotype distribution and no evidence of association with AMI based on HWE. The Hardy-Weinberg equilibrium results for the studied SNPs are summarized in Table 7.

**Table 7:** Hardy–Weinberg equilibrium analysis of genotype distributions in cases and controls.

SNP IDs	Study subjects	No. of homozygous wild genotypes	No. of heterozygous genotypes	No. of Homozygous mutant genotypes	No of major alleles	No. of minor alleles	p-value
**rs2060793**	**All subjects**	183	263	56	629	375	0.0099
	**Control**	86	126	39	298	204	0.6
	**Case**	97	137	17	331	171	0.00071
**rs12794714**	**All subjects**	93	187	80	373	347	0.53
	**Control**	51	90	39	192	168	1
	**Case**	42	97	41	181	179	0.37

### 3.15 Analysis of linkage disequilibrium in the study subjects

The linkage disequilibrium (LD) analysis between rs2060793 and rs12794714 suggests a weak linkage disequilibrium between these variants. The D statistic of 0.0987 indicates minimal deviation from expected haplotype frequencies under independence. The D’ statistic of 0.47 suggests moderate linkage disequilibrium, but it does not imply complete linkage between the variants. Additionally, the r² value of 0.11 demonstrates a weak association. The p-value of 0.054 is slightly above the conventional significance threshold of 0.05, indicating that the observed LD is not statistically significant. Overall, these results suggest that rs2060793 and rs12794714 are not strongly linked in the Bangladeshi population Table 8.

**Table 8:** Linkage disequilibrium analysis between rs2060793 and rs12794714.

Variant ID	D Statistic	D’ Statistic	r² Statistic	p-value
**rs2060793**	0.0987	0.47	0.11	0.054
**rs12794714**				

### 3.16 Combined effect of both SNPs on AMI risk

The combined effect of rs12794714 and rs2060793 on AMI risk using a logistic regression model using RStudio with age and gender as covariates is given in the S4 Table. According to the result, none of the combined genotypes showed a statistically significant association with AMI compared to the reference genotype (rs12794714 CC + rs2060793 TT). ORs have indicated trends of increased or decreased risk for certain genotype combinations, but none of them reach significance.

### 3.17 Possible regulatory mechanisms

The variant rs2060793 may have regulatory potential, according to its RegulomeDB (https://regulomedb.org/regulome-search/) rank of 1f, indicating possible transcription factor binding and chromatin activity. Moreover, this variant may reside in an active enhancer region in the liver, suggesting its role in modulating gene expression in hepatic tissue. The score of 0.55436 further supports its possible functional relevance. As rs2060793 showed a significant association with acute myocardial infarction in our study, we hypothesized that it may influence gene expression by altering transcription factor binding. To explore this possibility, we utilized motif prediction tool JASPAR (https://jaspar.elixir.no/). Prediction of transcription factor binding sites encompassing the variant position (rs2060793) using JASPAR is given in S5 Table. The JASPAR transcription factor binding analysis for rs2060793 suggests the polymorphism position might have regulatory role, with notable binding predictions for CRX (score: 11.73, probability: 0.97) and HNF4α (score: 10.13, probability: 0.87). Although the JASPAR relative score for CRX was higher than that for HNF4α, HNF4α was chosen for this study for its role in liver-specific gene regulation, aligning with the hepatic expression and function of *CYP2R1*, the target gene of interest.

### 3.18 Docking analysis of HNF4α with wild-type and rs2060793 mutant DNA

**Fig 11:**
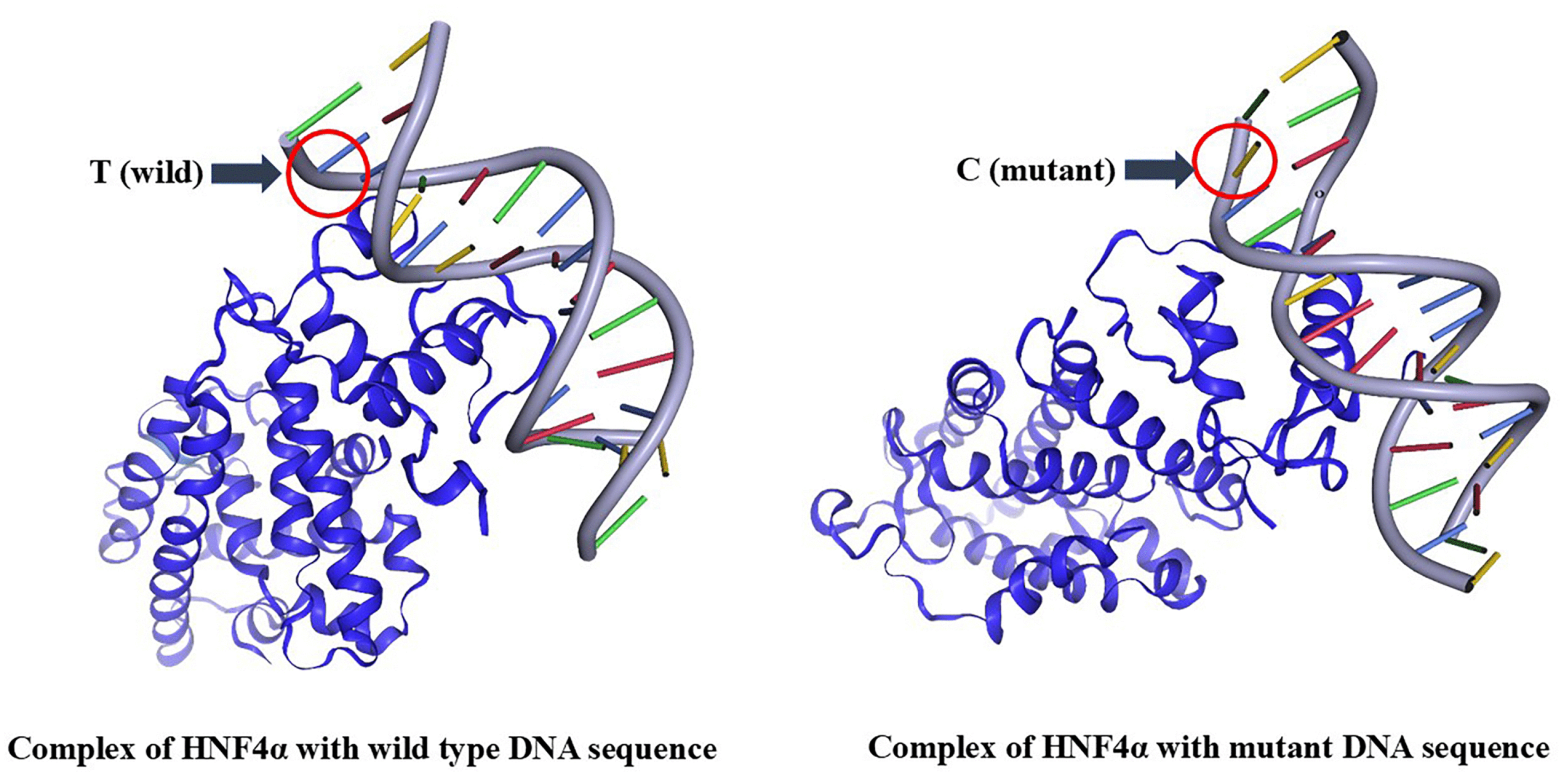
Molecular docking of wild-type (left) and rs2060793 mutant DNA with HNF4α protein (right).

Docking of HNF4α (PDB ID: 4IQR) with wild-type and rs2060793 mutant DNA was performed using HADDOCK 2.4. The DNA binding site was predicted by JASPAR, and the HNF4α binding region (amino acids 57–132) was retrieved from UniProt. HADDOCK scores were calculated as −100.0 + /- 4.6 for the wild-type DNA and −103.7 + /- 12.9 for the rs2060793 mutant DNA. Visualization of molecular docking complex of wild-type and rs2060793 mutant DNA with HNF4α protein is given in Fig 11.

Scores derived from DNAproDB are given in Table 9 which predicted the interaction differences between HNF4α and two DNA variants—wild-type and rs2060793 mutant DNA. In the wild-type complex, HNF4α is predicted to form 51 nucleotide-residue (Nuc-Res) interactions, including 9 weak contacts, supported by a buried accessible surface area (BASA) of 1166.96 Å². The interaction is predicted to be stabilized by 20 hydrogen bonds, 122 van der Waals (vdW) contacts, and a hydrophobicity score (SAP) of −1.8115. DNAproDB predicted the involved protein region is a helix, consistent with HNF4α’s typical DNA-binding domain.

**Table 9:** Comparison of HNF4α protein interactions with wild-type and rs2060793 mutant DNA using DNAproDB analysis.

Parameter	HNF4α- wild type DNA	HNF4α- rs2060793 mutant DNA
**Nuc-Res Interactions**	51	41
**Weak Nuc-Res Interactions**	9	10
**Total BASA [Å²]**	1166.963	1040.183
**Total Hbonds**	20	23
**Total vdW**	122	124
**Hydrophobicity Score (SAP)**	−1.6155	−1.6690
**Secondary Structure Composition**	Helix	Irregular

In the mutant complex, the analysis predicts a notable loss of 10 Nuc-Res interactions (41 total, with 10 weak), indicating possible reduced contact and potentially weaker binding. This is reflected in predicted lower BASA of 1040.18 Å² (∼11% decrease). Although hydrogen bonds may slightly increase to 23 and vdW contacts are predicted to remain similar (124), the SAP score of −1.5343 suggests a slightly less hydrophobic interface. Additionally, the interacting protein segment is predicted to adopt an irregular structure, possibly suggesting conformational changes induced by the mutation.

## 4. Discussion

This study aimed to elucidate the relationship between two functional polymorphisms in the *CYP2R1* gene (rs2060793 and rs12794714) and acute myocardial infarction (AMI) in a Bangladeshi population. *CYP2R1* encodes a 25-hydroxylase enzyme, crucial for converting vitamin D into its primary circulating form 25(OH)D, and genetic variants in this gene have been implicated in altered vitamin D metabolism and associated disease risk [27,55].

Demographic analysis showed no significant differences in age or sex distribution between AMI patients and controls, minimizing potential confounding. However, unfavorable lipid profiles, characterized by higher LDL, total cholesterol, and triglycerides, and lower HDL levels were observed in AMI patients compared to healthy subjects. These findings align with previous studies indicating that dyslipidemia is common in AMI patients [56,57]. Notably, AMI patients had markedly elevated levels of hsTroponin I (3918 ± 471 pg/mL in cases vs. 1.845 ± 0.079 pg/mL in controls, p < 0.0001). These findings align with established diagnostic criteria for AMI, where elevated troponin levels are a hallmark of myocardial infarction [2].

Both polymorphisms did not show a significant association with lipid parameters, including HDL, LDL, triglycerides, and total cholesterol. For rs2060793, a modest trend toward elevated LDL levels was observed among TC carriers, triglyceride levels demonstrated a gradual increasing pattern across genotypes and total cholesterol levels were modestly higher in heterozygous carriers. However, the association did not reach statistical significance. These findings suggest that the contribution of these variants to AMI susceptibility may not be mediated through alterations in lipid metabolism. Instead, their potential role may involve alternative biological pathways, such as modulation of vitamin D metabolism, inflammatory responses, endothelial function, or gene–environment interactions. It is also possible that the effect size of these variants on lipid traits is small and requires a larger sample size to detect statistically significant differences. Further large-scale and functional studies are needed to clarify the mechanistic role of these polymorphisms in cardiovascular risk.

Our results revealed that AMI patients had significantly lower circulating vitamin D compared to controls (p < 0.0001). These findings align with prior studies that have identified vitamin D deficiency as an independent cardiovascular risk factor [58]. Vitamin D plays a protective role in cardiovascular health by suppressing the renin–angiotensin–aldosterone system (RAAS), improving endothelial function, modulating vascular smooth muscle proliferation, and reducing systemic inflammation [10,12]. Deficiency may therefore accelerate the pathogenesis of AMI. A significant but weak negative correlation was observed between serum vitamin D levels and LDL cholesterol, triglycerides, and total cholesterol, indicating that lower vitamin D levels were associated with higher levels of these atherogenic lipid parameters. However, no significant association was found between vitamin D and HDL cholesterol. These findings suggest that vitamin D deficiency may contribute to an adverse lipid profile, which aligns with previous studies [59].

Although vitamin D levels across rs2060793 genotypes did not reach statistical significance, a decreasing trend was observed toward the homozygous mutant genotype. Notably, individuals carrying the homozygous mutant genotype exhibited comparatively lower vitamin D levels. This directional pattern is biologically relevant, as vitamin D deficiency has been widely implicated in endothelial dysfunction, inflammation, and increased cardiovascular risk [10,12]. The observed trend suggests that rs2060793 may contribute to AMI risk, potentially through modulation of vitamin D status or related downstream pathways. Even in the absence of statistical significance for vitamin D levels, the consistent decrease in the mutant genotype supports a possible functional effect. It is plausible that rs2060793 influences local tissue-level vitamin D activity, gene regulation, or inflammatory signaling rather than markedly altering circulating vitamin D concentrations. On the other hand, while rs12794714 may influence the measured vitamin D level without contributing substantially to AMI susceptibility. Rather, it might have a protective role as the mutant genotype significantly increased vitamin D level or it might have effect solely on vitamin D level.

Males exhibit a more pronounced difference (24.27 ± 1.159 ng/mL in cases vs. 31.09 ± 1.006 ng/mL in controls, p < 0.0001) than females (23.05 ± 1.563 ng/mL vs. 28.28 ± 1.627 ng/mL, p = 0.0149). The gender disparity may be explained by lifestyle and cultural differences influencing sun exposure, as well as hormonal effects on vitamin D homeostasis [60]. Younger individuals may be more sensitive to vitamin D deficiency due to fewer accumulated comorbidities and a stronger physiological response to micronutrient imbalances [61].

From HPLC analysis, vitamin D_3_ eluted at approximately 6 minutes, whereas earlier peaks (2–5 minutes) are more plausibly attributable to previtamin D_3_, photochemical isomers (e.g., lumisterol or tachysterol), minor degradation products, or matrix-related components [62]. As vitamin D_3_ is the predominant endogenous form synthesized in human skin whereas vitamin D_2_ is mainly derived from plant sources and fortified foods, the study focused on endogenous vitamin D status in relation to disease. That’s why, 25-hydroxyvitamin D_3_ [25(OH)D_3_] was considered the most biologically relevant analyte to represent total 25(OH)D. Therefore, the calibration standard contained only vitamin D_3_. Minor retention time shifts observed between standard and sample chromatograms may be attributed to matrix effects, slight variations in mobile phase composition, column equilibration differences, or temperature fluctuations [63]. However, these shifts remained within acceptable analytical limits, and peak identity was confirmed based on consistent chromatographic behavior and validated method parameters. Only the peak corresponding to the retention time of the 25(OH)D_3_ standard was integrated for quantitative analysis.

The present study investigated the association of two polymorphisms in the *CYP2R1* gene—rs2060793 and rs12794714—with acute myocardial infarction (AMI), revealing a sex-specific pattern in genetic susceptibility. Notably, a strong and statistically significant association between rs2060793 and AMI was observed in males under both co-dominant and dominant models, whereas no such association was found in females. These findings are consistent with prior studies conducted in different populations. For example, Sedky et al. reported that male carriers of the variant genotypes (homozygous and heterozygous mutant) of rs2060793 had significantly increased odds of myocardial infarction among Egyptian patients, with odds ratios comparable to those reported in the present analysis [26]. Similarly, a study by Wang et al. in a Chinese Han population identified a significant association of rs2060793 with coronary heart disease risk specifically in males, further supporting a potential sex-dependent genetic effect of this polymorphism in cardiovascular pathology [27].

In contrast, rs12794714 did not show a statistically significant association with AMI in either sex across all genetic models, despite a trend toward increased risk in individuals carrying the variant genotypes. This observation also aligns with previous studies. For instance, Sedky et al. (2014) found no significant association between rs12794714 and coronary artery disease (CAD) among Egyptian men, suggesting that this particular SNP may not play a critical role in cardiovascular risk, at least in certain ethnic or demographic contexts [26]. On the other hand, in Chinese rural population, rs12794714 was reported to interact with general and central obesity, such that individuals carrying the mutant genotype and obesity had significantly higher odds of hypertension [64] Moreover, obesity is one of the major predisposition in causing cardiovascular diseases including AMI [65] Thus, it can be said that genetic associations found in different populations may not align identically in Bangladesh due to difference in lifestyles and many other geographical factors [66].

As rs2060793 and rs12794714 polymorphisms showed no significant overall association with AMI in female individuals, it can be due to splitting the cohort by sex which in turn reduce sample size and statistical power. This result could also reflect the effect of estrogen and other female hormones on expression and activity of vitamin D pathway genes including *CYP2R1*. If hormonal regulation or menstrual/menopausal status strongly affects vitamin D metabolism, a genetic variant’s influence on AMI risk may be blunted or different in direction in females [67]. Similarly, environmental effects may play a role which causes female patients (23.05 ± 1.563 ng/mL) and controls (28.28 ± 1.627 ng/mL) having lower vitamin D levels than male patients (24.27 ± 1.159 ng/mL) and controls (31.09 ± 1.006 ng/mL) respectively, potentially attenuating the observable genotypic effect on AMI risk. Sex-stratified comparisons were exploratory, that’s why these results should be interpreted as hypothesis-generating rather than confirmatory. Larger studies are required to validate these observations.

Age-stratified analysis revealed a marked association between both *CYP2R1* polymorphisms—rs2060793 and rs12794714—and acute myocardial infarction (AMI) among participants aged 60 years or younger, particularly under the co-dominant and dominant genetic models. These findings are in line with previous research suggesting that genetic susceptibility to cardiovascular events may be more pronounced in younger individuals due to a stronger influence of genetic predisposition in the absence of age-related confounders such as prolonged comorbidity exposure or polypharmacy [28]. On the other hand, no significant associations were found in the > 60 years age group for both SNPs. In a study it was revealed that, genetic risk for coronary artery disease (CAD) has been shown to be stronger for early‑onset disease than late‑onset disease [68]. This study compared early vs late onset CAD, people with early onset had higher genetic risk scores than those with later onset [68]. Moreover, in older individuals the reason of insignificant genotype association with AMI might be due to other causes of disease become more prominent in old age. However, these findings should not be considered definitive or confirmatory, but rather interpreted as hypothesis-generating observations requiring validation in larger independent studies. The analytical framework distinguished between pre-specified primary analyses and exploratory secondary analyses. The primary analysis evaluating association between SNPs and AMI was pre-specified based on prior evidence of involvement of the polymorphism in disease susceptibility. Analyses under different inheritance models and age- and sex-stratified analyses were exploratory and performed to investigate potential effect modification. Conservative adjustments may reduce the ability to detect true effects which can obscure biologically meaningful associations in exploratory candidate-gene research [69].

Age- and gender-adjusted logistic regression analysis demonstrated that the association between rs2060793 and AMI risk remained significant after controlling for these covariates. In the co-dominant model, both TC (OR = 2.71, 95% CI: 1.42–5.17) and CC (OR = 3.19, 95% CI: 1.63–6.24) genotypes showed a significantly increased risk compared with the TT genotype (p = 0.0018). Similarly, the dominant model (TC + CC vs TT) demonstrated a strong association (OR = 2.89, 95% CI: 1.55–5.40; p = 5.00 × 10 ⁻ ⁴). In contrast, rs12794714 showed no significant association with AMI after adjustment in any genetic model (all p > 0.05). There is no significant multicollinearity among predictor variables, with all adjusted GVIF values close to 1, supporting the stability of the regression models. The Hosmer–Lemeshow test indicated acceptable fit for the rs2060793 model. In contrast, the rs12794714 model showed lack of fit, which may reflect the absence of a strong association with MI risk, limited predictive contribution of the variant, or the influence of unmeasured confounding factors not included in the model. Although age and gender adjustments were performed, the potential influence of residual confounding from other cardiovascular risk factors such as lipid parameters, lifestyle, dietary habits, smoking status and vitamin D levels were not included in the regression models. Since these variables may function as intermediates in the biological pathway linking *CYP2R1* polymorphisms to AMI, adjusting for them could potentially attenuate the genetic effect. Nevertheless, the lack of multivariable adjustment for these factors may be considered a limitation of the present study and larger studies incorporating additional covariates are warranted to validate these findings.

From Hardy- Weinberg equilibrium (HWE) analysis, we observed that rs2060793 had a significant association with AMI. The frequency of the minor allele was markedly higher in cases compared to controls (p = 0.00071 in cases vs. p = 0.6 in controls). While deviation in cases may reflect true genetic association with disease susceptibility, alternative explanations such as genotyping error or population stratification must be considered. Given that strict quality control measures were implemented and controls were in HWE, technical error is less likely. However, the possibility of residual population substructure cannot be completely excluded and represents a limitation of the study. In contrast, rs12794714 showed no significant association with AMI either in overall subjects (p = 0.53) or in stratified case–control groups (p = 1 in controls; p = 0.37 in cases).

Linkage disequilibrium (LD) analysis indicated that rs2060793 and rs12794714 are weakly linked (r² = 0.11). An r² of 0.11 means only 11% of the variance at one SNP can be predicted by the other. The D’ statistic of 0.47 suggests moderate linkage disequilibrium; however, this value does not imply complete linkage. The D statistic of 0.0987 reflects a low degree of non-random association between the two SNPs. These results are consistent with findings from Manousaki et al., who documented similarly low correlation between certain *CYP2R1*-region SNPs across diverse global populations in their genome-wide meta-analysis of vitamin D levels [55]. The p-value of 0.054, slightly above the conventional significance threshold of 0.05, indicates that the observed LD is not statistically significant. Because the LD is low, the two SNPs can reasonably be treated as largely independent in statistical models; that is, their effects are unlikely to be conflated. Combined genotype analysis did not reveal statistically significant effects beyond individual SNP associations. Low LD can be a reason for this outcome.

From in silico analyses, it was predicted that rs2060793 has transcriptional potential (RegulomeDB rank 1f), with the variant located in an active enhancer region of the liver, consistent with the hepatic expression of *CYP2R1*. Transcription factor binding site prediction by JASPAR suggested that HNF4α, a liver-enriched regulator, as a potential binding partner at this locus. HNF4α is a liver-enriched transcription factor regulating CYP gene expression [70]. Docking analysis further predicted a loss of 10-residue in nucleotide-protein interactions in mutant DNA complex and a predicted 11% reduction in buried accessible surface area compared to the wild-type, consistent with weakened binding affinity, accompanied by a shift from a stable helical binding region to an irregular conformation. These computational findings suggest possible reduced binding stability, potentially affecting *CYP2R1* transcriptional regulation. Together with the observed association between rs2060793 and AMI in our cohort, these results further strengthen the hypothesis that rs2060793 may contribute to disease risk by modulating HNF4α-mediated regulation of *CYP2R1* transcription. These observations, however, remain predictive and require experimental validation to confirm their biological significance.

Taken together, these results suggest that rs2060793 contributes to AMI pathogenesis through two potential mechanisms: genetic predisposition toward vitamin D deficiency and impaired transcriptional regulation. The age- and sex-specific effects further highlight the need for stratified risk assessment. Moreover, significant association of rs12794714 in younger individuals suggests a potentially distinct, developmentally timed mechanism of influence on cardiovascular risk.

These findings underscore the relevance of integrating genetic, biochemical, and demographic factors in cardiovascular risk assessment and may inform future strategies for personalized prevention and intervention.

## Supporting information

S1 Fig Uncropped raw gel images. (PDF)

S1 Table The primers for PCR targeting CYP2R1 gene polymorphisms. (PDF)

S2 Table Frequency distribution of CYP2R1 rs12794714 genotypes in male and female subjects. (PDF)

S3 Table Individual-Level genotype and vitamin D data for study participants. (PDF)

S4 Table Combined effect of both SNPs on AMI risk. (PDF)

S5 Table Prediction of transcription factor binding sites encompassing the variant position (rs2060793) using JASPAR. (PDF)
