# Supplementary material for: Genetic association and computational analysis of CYP2R1 gene polymorphisms rs2060793 and rs12794714 with vitamin D deficiency and acute myocardial infarction in the Bangladeshi population: A case control study
Source: PLoS One. 2026 Jun 5;21(6):e0350994. doi: 10.1371/journal.pone.0350994 (PMC13240929; doi:10.1371/journal.pone.0350994)
Supplement: S1 Fig — (PDF) [file pone.0350994.s001.pdf]

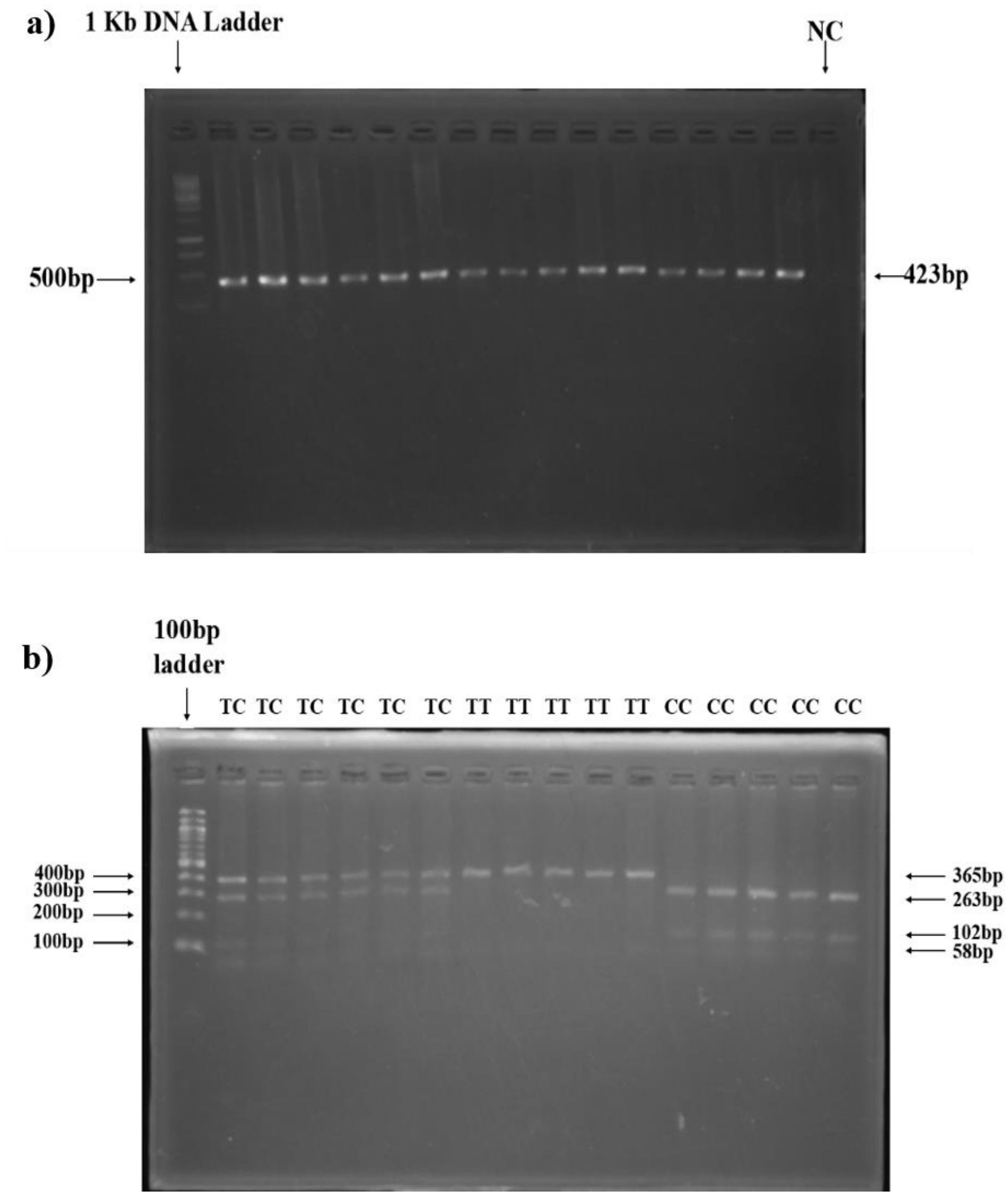

Figure 5: Visualization of PCR and restriction digested products (rs2060793). a) A 423 bp amplicon of the *CYP2R1* gene, targeting the rs2060793 polymorphism, **b)** Restriction digestion products of TT (365bp, 58bp), TC (365bp, 263bp, 102bp and 58bp) and CC (263bp, 102bp and 58bp) genotypes.

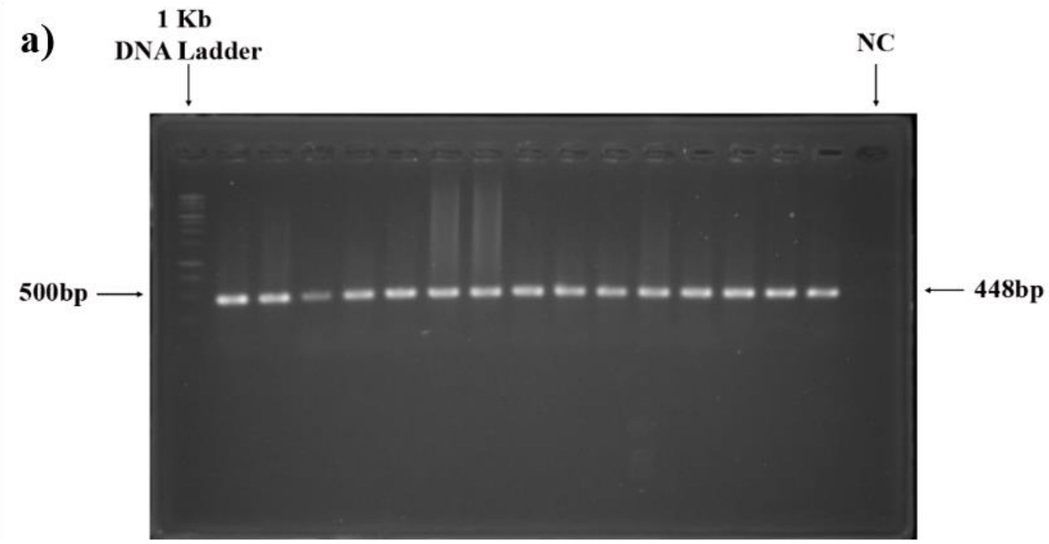

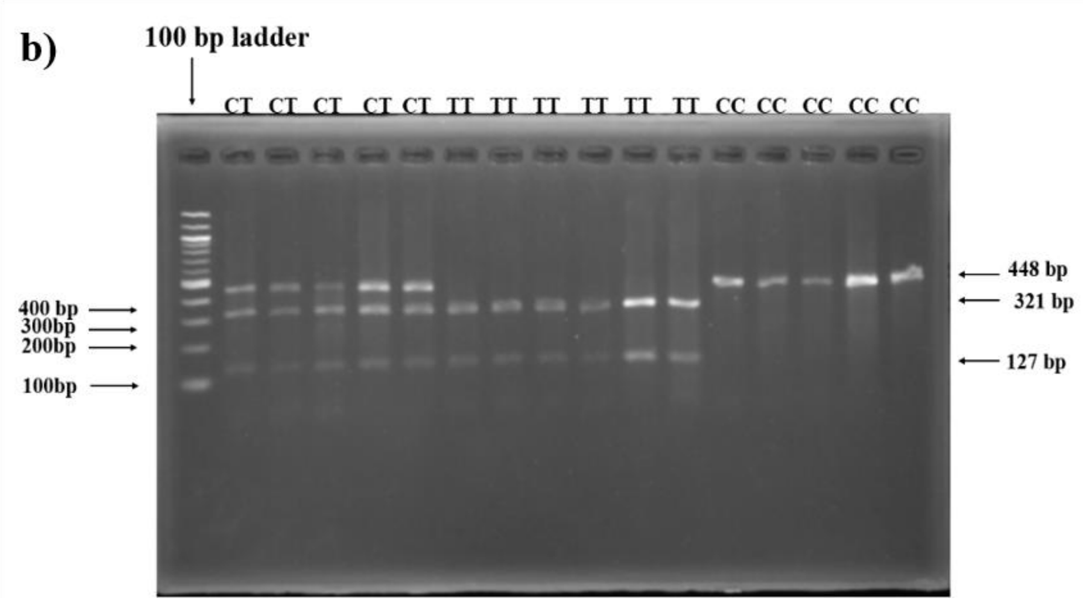

Figure 6: Visualization of PCR and restriction digested products (rs12794714). a) PCR product of the desired 448 bp *CYP2R1* gene segment amplicon targeting rs12794714, **b)** Restriction digestion products of CC (448bp), CT (448bp, 321bp and 127bp) and TT (321bp and 127bp) genotypes.
