# Supplementary material for: Genetic association and computational analysis of CYP2R1 gene polymorphisms rs2060793 and rs12794714 with vitamin D deficiency and acute myocardial infarction in the Bangladeshi population: A case control study
Source: PLoS One. 2026 Jun 5;21(6):e0350994. doi: 10.1371/journal.pone.0350994 (PMC13240929; doi:10.1371/journal.pone.0350994)
Supplement: S1 Table — (PDF) [file pone.0350994.s002.pdf]

S1 Table: The primers for PCR targeting CYP2R1 gene polymorphisms

Variant ID	Primer Sequences	Amplicon Size(bp)
rs2060793	F: 5’ GCGTGGGAGATGGAGAGAAA 3’<br>R: 5’ ATTCCTTGGCTTTCGTCCCC 3’	423 bp
rs12794714	F: 5’ CGCTCTTCCTGCTGCTCTT 3’<br>R: 5’ GAGCGGTAGCGGGAAAATCA 3’	448 bp
