# Supplementary material for: Genetic association and computational analysis of CYP2R1 gene polymorphisms rs2060793 and rs12794714 with vitamin D deficiency and acute myocardial infarction in the Bangladeshi population: A case control study
Source: PLoS One. 2026 Jun 5;21(6):e0350994. doi: 10.1371/journal.pone.0350994 (PMC13240929; doi:10.1371/journal.pone.0350994)
Supplement: S2 Table — (PDF) [file pone.0350994.s003.pdf]

S2 Table: Frequency distribution of CYP2R1 rs12794714 genotypes in male and female subjects

SNPs	Genetic Model	Genotype Status	Control n (%)	Case n (%)	p-value	Odds Ratio	95% CI
Male (n=180)	Co-dominant Model	CC	51 (28.3%)	42 (23.3%)	0.57	1(Ref.)	
		CT	90 (50%)	97 (53.9%)		1.31	0.79 to 2.16
		TT	39 (21.7%)	41 (22.8%)		1.28	0.7 to 2.32
	Dominant Model	CC	51 (28.3%)	42 (23.3%)	0.28	1(Ref.)	
		CT+TT	129 (71.7%)	138 (76.7%)		1.30	0.81 to 2.09
	Recessive Model	CC+CT	141 (78.3%)	139 (77.2%)	0.8	1((Ref.)	
		TT	39 (21.7%)	41 (22.8%)		1.07	0.65 to 1.75
Female (n=71)	Co-dominant Model	TT	12 (16.9%)	8 (11.3%)	0.61	1(Ref.)	
		TC	35 (49.3%)	36 (50.7%)		1.54	0.56 to 4.23
		CC	24 (33.8%)	27 (38%)		1.69	0.59 to 4.82
	Dominant Model	TT	12 (16.9%)	8 (11.3%)	0.33	1(Ref.)	
		TC+CC	59 (83.1%)	63 (88.7%)		1.6	0.61 to 4.19
	Recessive Model	TT+TC	47 (66.2%)	44 (62%)	0.6	1(Ref.)	
		CC	24 (33.8%)	27 (38%)		1.2	0.60 to 2.39

$p<0.05$ was considered as level of significance. Result is represented as Mean± SEM
