# Supplementary material for: Genetic association and computational analysis of CYP2R1 gene polymorphisms rs2060793 and rs12794714 with vitamin D deficiency and acute myocardial infarction in the Bangladeshi population: A case control study
Source: PLoS One. 2026 Jun 5;21(6):e0350994. doi: 10.1371/journal.pone.0350994 (PMC13240929; doi:10.1371/journal.pone.0350994)
Supplement: S3 Table — (PDF) [file pone.0350994.s004.pdf]

S3 Table: Individual-Level genotype and vitamin D data for study participants

Subjects	Group	Genotype (rs12794714)	Genotype (rs2060793)	Level of Vitamin D (ng/mL)
S1	Control	TT	TC	27.56013777
S2	Control	TT	TC	33.55228034
S3	Control	CT	TC	22.15383046
S4	Control	CT	TC	48.8729131
S5	Control	CC	TC	29.01968115
S6	Control	CT	TC	29.10432134
S7	Control	TT	CC	44.92706979
S8	Control	TT	TC	67.67026693
S9	Control	CT	TC	16.19629568
S10	Control	CT	TC	25.01151535
S11	Control	CC	TC	44.98435698
S12	Control	CC	TC	47.40667798
S13	Control	CC	TC	39.43991376
S14	Control	TT	CC	48.41068584
S15	Control	CC	CC	40.47761604
S16	Control	CC	TT	29.13033461
S17	Control	CC	TC	73.6437596
S18	Control	CT	TT	37.99093349
S19	Control	CC	TT	43.63160801
S20	Control	CT	TT	47.51567645
S21	Control	TT	TC	38.82481811
S22	Control	CC	TT	38.08317502
S23	Control	TT	TT	30.68778459
S24	Control	CT	TT	29.51008646
S25	Control	CC	TT	29.97836208
S26	Control	CT	TC	26.30030959
S27	Control	TT	CC	19.67664457
S28	Control	CT	TC	25.01853098
S29	Control	CC	TT	21.70436211

S30	Control	CC	TT	24.70037077
S31	Control	CT	TC	22.36889191
S32	Control	CT	TT	29.09233039
S33	Control	CC	TT	18.1318293
S34	Control	CC	TT	16.17871151
S35	Control	CT	TT	10.85753408
S36	Control	CC	TC	29.52561869
S37	Control	CC	TC	27.96942683
S38	Control	CT	TT	20.49563581
S39	Control	CC	TT	32.9547163
S40	Control	CC	CC	21.03368156
S41	Control	CC	TC	15.92502137
S42	Control	TT	TT	20.21441273
S43	Control	TT	TT	30.8171173
S44	Control	CC	CC	49.36201912
S45	Control	CT	CC	30.33991421
S46	Control	CT	TT	22.35070958
S47	Control	CC	TC	24.72884706
S48	Control	CT	TT	32.36464991
S49	Control	CT	TC	24.73053985
S50	Control	CC	TC	23.40807538
S51	Control	CT	CC	28.96380143
S52	Control	CC	TC	25.60623144
S53	Control	CT	CC	24.28646892
S54	Control	CT	CC	26.36401393
S55	Control	CT	CC	16.52065442
S56	Control	CC	CC	27.88498281
S57	Control	CC	CC	22.68829032
S58	Control	CC	CC	25.70620352
S59	Control	CT	CC	22.45071421
S60	Control	TT	TC	23.01496561
S61	Control	TT	TC	28.3109791
S62	Control	CT	CC	22.19438724

S63	Control	CT	TC	28.35427538
S64	Control	TT	CC	23.98045436
S65	Control	CT	TC	14.67014517
S66	Control	CC	TT	26.40620866
S67	Control	CC	CC	47.09622262
S68	Control	CT	TC	21.38099886
S69	Control	CC	CC	70.55163635
S70	Control	CC	TC	16.45412778
S71	Control	CC	CC	18.22184399
S72	Control	TT	CC	24.4553945
S73	Control	CC	CC	10.74152767
S74	Control	CC	CC	14.22960856
S75	Control	CT	CC	22.46596171
S76	Control	CC	CC	17.69891596
S77	Control	CT	CC	29.47582664
S78	Control	CT	CC	27.98820866
S79	Control	TT	CC	16.88861716
S80	Control	CC	CC	25.40067032
S81	Control	TT	TC	48.3663578
S82	Control	CT	TT	18.98230957
S83	Control	CC	CC	23.09952519
S84	Control	TT	CC	39.04164365
S85	Control	CT	CC	26.73620633
S86	Control	CT	TC	19.80661617
S87	Control	TT	TC	76.38520569
S88	Control	CT	TT	32.02838055
S89	Control	CT	CC	67.02495003
S90	Control	CT	CC	31.88153509
S91	Control	CT	CC	27.3167361
S92	Control	CT	TT	32.97028606
S93	Control	CT	CC	27.3167361
S94	Control	TT	CC	32.97028606
S95	Control	CT	TC	15.95651889

S96	Control	TT	CC	53.56148924
S97	Control	CT	TC	45.37624789
S98	Control	TT	CC	52.70941237
S99	Control	CT	TC	31.36855089
S100	Control	CT	CC	28.31975294
S101	Control	CT	CC	37.89891194
S102	Control	CT	TC	6.452119605
S103	Control	CT	TC	3.490699153
S104	Control	CT	TC	41.85641362
S105	Control	CC	TT	40.23498579
S106	Control	CC	TC	19.98203966
S107	Control	CT	CC	22.30092769
S108	Control	CT	TC	24.45439176
S109	Control	CT	TC	12.14285445
S110	Control	CT	TT	32.06057959
S111	Control	CT	CC	24.97425586
S112	Control	CT	TC	42.30789654
S113	Control	CT	CC	8.035220553
S114	Control	CT	TC	13.75659462
S115	Control	TT	CC	43.72646485
S116	Control	CT	TC	18.46821122
S117	Control	TT	CC	38.65113279
S118	Control	CT	TC	25.44133693
S119	Control	CC	TC	18.0736337
S120	Control	TT	TT	42.17812549
S121	Control	CC	TC	40.19094886
S122	Control	CT	TC	26.73991089
S123	Control	TT	CC	17.12261998
S124	Control	CT	TC	28.2248549
S125	Control	CT	TC	12.31170444
S126	Control	CT	TC	19.67277846
S127	Control	CC	CC	34.2492563
S128	Control	CT	TC	43.77389084

S129	Control	CC	CC	33.12452411
S130	Control	CT	TC	22.29266114
S131	Control	CC	TT	49.72771362
S132	Control	CC	TC	66.42868564
S133	Control	CC	TC	29.22384224
S134	Control	CC	TC	26.10521623
S135	Control	CC	CC	28.70491235
S136	Control	CT	TC	30.46558339
S137	Control	CT	TC	28.63152326
S138	Control	CC	TC	27.18872625
S139	Control	CT	TC	24.23770123
S140	Control	CT	TC	26.95134972
S141	Control	CC	TC	28.63152326
S142	Control	CT	CC	24.37937432
S143	Control	CT	CC	28.27738616
S144	Control	CT	TC	26.27330467
S145	Control	CC	TT	31.27638027
S146	Control	CT	CC	32.20602844
S147	Control	CT	TC	34.65492787
S148	Control	CT	TC	24.81026063
S149	Control	TT	CC	23.60855698
S150	Control	CC	TC	22.514283
S151	Control	CT	CC	28.00264591
S152	Control	CC	TT	26.83933309
S153	Control	CC	TT	20.15617246
S154	Control	CT	TC	22.29825405
S155	Control	CT	TC	25.70078749
S156	Control	CT	CC	25.20429688
S157	Control	CC	TC	17.41300518
S158	Control	TT	TC	22.47669111
S159	Control	CT	CC	24.50436123
S160	Control	CT	TC	15.49238096
S161	Control	CT	TC	12.23835307

S162	Control	CT	CC	24.08922924
S163	Control	CT	TC	20.46143124
S164	Control	CC	CC	18.83179355
S165	Control	CT	CC	18.14912308
S166	Control	CT	CC	11.82705893
S167	Control	CC	TC	14.12928522
S168	Control	CT	CC	17.9212007
S169	Control	TT	CC	23.76944973
S170	Control	CT	TT	13.19953378
S171	Control	CC	CC	12.217114
S172	Control	CC	CC	16.03600097
S173	Control	CC	TC	18.85059525
S174	Control	CT	CC	12.86731034
S175	Control	CT	CC	10.35289492
S176	Control	CC	TC	24.2121084
S177	Control	CT	TT	17.44741521
S178	Control	CC	TC	20.82392393
S179	Control	CT	CC	48.70435202
S180	Control	CT	CC	20.84695612
S181	Control	CC	CC	23.81775877
S182	Control	CT	TC	25.80029039
S183	Control	CT	CC	24.08013167
S184	Control	CC	CC	24.40850567
S185	Control	TT	CC	40.20502322
S186	Control	CT	TC	22.22040953
S187	Control	CC	CC	24.04888986
S188	Control	TT	TC	50.37382425
S189	Control	CT	CC	34.20447857
S190	Control	CC	TC	30.65218878
S191	Control	CC	TC	42.4711628
S192	Control	CC	CC	48.26265806
S193	Control	TT	CC	15.73167067
S194	Control	TT	TC	47.76419402

S195	Control	CC	TC	51.09093313
S196	Control	TT	CC	36.74013054
S197	Control	CT	TC	42.74377977
S198	Control	TT	TC	22.2479216
S199	Control	TT	TC	43.48622976
S200	Control	CT	CC	45.00903768
S201	Control	CT	TC	34.76506536
S202	Control	CC	TT	40.07899188
S203	Control	CT	TC	23.27392525
S204	Control	CT	TC	13.01246609
S205	Control	CT	TC	46.89927683
S206	Control	CC	TC	42.79264259
S207	Control	CT	TC	54.76724924
S208	Control	CT	TC	33.59206081
S209	Control	CT	TC	35.2289976
S210	Control	TT	TC	35.33659262
S211	Control	CT	TC	38.73697517
S212	Control	TT	TC	32.9002506
S213	Control	TT	TC	16.20660507
S214	Control	TT	CC	50.92180593
S215	Control	CT	TC	61.05155823
S216	Control	CC	TC	21.03345607
S217	Control	TT	CC	30.06820856
S218	Control	CT	TC	37.71486318
S219	Control	TT	TC	25.41597899
S220	Control	CT	TC	19.27532902
S221	Control	CC	TC	38.32211699
S222	Control	TT	CC	65.62658532
S223	Control	CC	TC	51.59114736
S224	Control	CT	TT	45.33109812
S225	Control	CT	TC	37.75817255
S226	Control	CT	TC	47.11049995
S227	Control	TT	TC	35.84349678

S228	Control	CC	TC	18.33297032
S229	Control	CT	CC	50.83499723
S230	Control	CT	TT	46.37234113
S231	Control	CC	TC	48.56983034
S232	Control	CT	TC	69.73763773
S233	Control	CC	TC	35.63853709
S234	Control	TT	TC	16.02251311
S235	Control	CT	TC	37.63274905
S236	Control	TT	CC	16.05502228
S237	Control	TT	TT	23.74026457
S238	Control	TT	TC	21.6206291
S239	Control	CT	CC	17.15095771
S240	Control	CT	TC	14.55272114
S241	Control	CT	TC	37.06022957
S242	Control	CT	TC	34.77574171
S243	Control	TT	TC	36.74403861
S244	Control	CT	TT	74.96640294
S245	Control	TT	TC	73.05878258
S246	Control	CT	CC	32.23232026
S247	Control	TT	TC	13.38377519
S248	Control	CT	TC	22.03974766
S249	Control	CT	TT	15.55615044
S250	Control	CC	TC	29.20742127
S251	Control	TT	TC	30.44640574
S252	Case	TT	CC	28.2
S253	Case	TT	TC	11.1
S254	Case	CT	CC	18.2
S255	Case	TT	CC	23
S256	Case	CT	CC	34.9
S257	Case	CC	CC	20.8
S258	Case	CC	TC	6.9
S259	Case	CT	CC	19.2
S260	Case	CT	TC	23.2

S261	Case	CT	TC	11.7
S262	Case	CT	CC	
S263	Case	TT	CC	8.4
S264	Case	TT	CC	4.7
S265	Case	CT	TC	32.3
S266	Case	CT	TC	35.5
S267	Case	TT	CC	3.9
S268	Case	CC	TC	5.4
S269	Case	CT	TC	8.4
S270	Case	CC	TC	11.4
S271	Case	CT	TC	20.6
S272	Case	CC	CC	14.3
S273	Case	CT	TC	44
S274	Case	CT	TC	39.3
S275	Case	CT	CC	27.5
S276	Case	CT	CC	22.3
S277	Case	CC	TC	29.3
S278	Case	CC	TT	12.2
S279	Case	CT	TC	16.1
S280	Case	CT	TC	41.4
S281	Case	CC	CC	18.3
S282	Case	CC	TC	21.5
S283	Case	CC	TC	23.9
S284	Case	CT	TC	11.1
S285	Case	CT	TC	24.3
S286	Case	CT	TT	7.8
S287	Case	CT	TC	16.9
S288	Case	CT	TC	32.8
S289	Case	TT	CC	41.3
S290	Case	CC	CC	10.2
S291	Case	CT	TC	33.5
S292	Case	CT	TC	

S293	Case	CT	CC	18.1
S294	Case	CT	TC	14.2
S295	Case	CC	TC	38.4
S296	Case	CC	TC	4.6
S297	Case	CT	TC	6
S298	Case	CC	TC	5.9
S299	Case	CC	TC	27.9
S300	Case	TT	TC	21.8
S301	Case	CT	TC	8.5
S302	Case	CC	TC	5.3
S303	Case	CT	TC	38
S304	Case	CT	TC	31.3
S305	Case	CC	TC	8.2
S306	Case	CC	TC	15.3
S307	Case	TT	TC	8.8
S308	Case	TT	CC	31.8
S309	Case	TT	CC	44.9
S310	Case	CT	CC	13.1
S311	Case	TT	TC	17.3
S312	Case	TT	TC	37.2
S313	Case	CT	TC	35.8
S314	Case	CC	CC	15.5
S315	Case	TT	TC	21.1
S316	Case	CC	CC	25.3
S317	Case	CC	TC	9.2
S318	Case	CT	TC	11.4
S319	Case	CT	CC	9.5
S320	Case	CT	TC	40.7
S321	Case	CT	TC	11.1
S322	Case	CT	TC	8.6
S323	Case	CC	TC	11.3
S324	Case	CT	TC	27.2
S325	Case	CT	TC	8.4

S326	Case	CT	TC	4.8
S327	Case	CT	TT	14.7
S328	Case	TT	CC	11
S329	Case	TT	CC	9.8
S330	Case	TT	CC	4.2
S331	Case	CC	CC	4
S332	Case	CT	CC	8.3
S333	Case	CT	CC	17.5
S334	Case	CC	TT	7.2
S335	Case	CT	CC	8.2
S336	Case	CC	TT	9.4
S337	Case	TT	CC	14.8
S338	Case	CT	TC	17.7
S339	Case	CT	TC	39.8
S340	Case	CT	TC	11.1
S341	Case	CC	TT	8.2
S342	Case	CC	CC	18.8
S343	Case	CT	TC	13
S344	Case	CT	CC	18.9
S345	Case	CC	TC	37
S346	Case	TT	CC	48.9
S347	Case	TT	CC	38.2
S348	Case	CC	TT	4.1
S349	Case	CT	TC	85.2
S350	Case	CT	TC	72.1
S351	Case	CT	CC	18.5
S352	Case	CT	CC	17.4
S353	Case	CC	CC	44.7
S354	Case	CT	CC	45.9
S355	Case	CT	TC	36.6
S356	Case	CC	TC	32.1
S357	Case	CT	TC	

S358	Case	CT	CC	10.4
S359	Case	CT	TC	10.1
S360	Case	CT	TC	33.9
S361	Case	TT	CC	6.6
S362	Case	TT	CC	40.4
S363	Case	CT	TC	37.9
S364	Case	CT	CC	23.2
S365	Case	TT	TT	31.8
S366	Case	TT	CC	30.6
S367	Case	CT	CC	31.7
S368	Case	CT	CC	11.9
S369	Case	CT	CC	12.9
S370	Case	CC	TT	82.1
S371	Case	CT	CC	12.8
S372	Case	CC	TT	16.8
S373	Case	CC	TT	31.7
S374	Case	CT	TC	36.6
S375	Case	TT	TC	23.7
S376	Case	CC	CC	10.2
S377	Case	CT	TC	34
S378	Case	CT	CC	46.3
S379	Case	TT	CC	33.4
S380	Case	CT	TC	11
S381	Case	TT	CC	43.2
S382	Case	CT	TC	31.5
S383	Case	CT	TC	38.6
S384	Case	CT	TC	13.3
S385	Case	CT	TC	33
S386	Case	CT	TT	15.4
S387	Case	TT	CC	14.6
S388	Case	CC	CC	13.1
S389	Case	CT	CC	13.5
S390	Case	TT	CC	38.7

S391	Case	CT	CC	38.1
S392	Case	CT	TC	10.1
S393	Case	CT	TC	44.3
S394	Case	CT	TC	9.4
S395	Case	CT	TC	22.6
S396	Case	TT	CC	79.1
S397	Case	CT	CC	8.7
S398	Case	CC	TC	39.1
S399	Case	CT	CC	18.9
S400	Case	CT	TC	20.3
S401	Case	CT	TC	8.3
S402	Case	CT	TC	31.6
S403	Case	TT	CC	39.4
S404	Case	CT	TC	4
S405	Case	CT	TC	8.5
S406	Case	TT	CC	10.1
S407	Case	TT	CC	
S408	Case	CC	TT	37.6
S409	Case	CT	CC	10.1
S410	Case	TT	CC	26.5
S411	Case	CT	CC	12.2
S412	Case	CC	TC	12.7
S413	Case	CC	TC	13.2
S414	Case	CC	CC	25.7
S415	Case	CT	CC	25.3
S416	Case	CT	TC	20.7
S417	Case	CC	TC	15.7
S418	Case	CT	CC	33.2
S419	Case	CT	CC	6.5
S420	Case	CT	TC	15.5
S421	Case	CT	CC	12.6
S422	Case	CT	CC	25.6

S423	Case	TT	TC	1.2
S424	Case	CT	TC	
S425	Case	CT	TC	47.6
S426	Case	CC	CC	14
S427	Case	CT	TC	
S428	Case	CT	TC	
S429	Case	CC	CC	21.9
S430	Case	CC	CC	19.5
S431	Case	CC	TC	18.8
S432	Case	CT	CC	36.9
S433	Case	CT	TC	17.5
S434	Case	TT	CC	14.6
S435	Case	CC	TC	15.9
S436	Case	CT	TC	13.9
S437	Case	CT	TT	37.3
S438	Case	TT	TC	22.7
S439	Case	CT	CC	30.5
S440	Case	CT	CC	
S441	Case	CT	TC	38
S442	Case	TT	CC	26.5
S443	Case	TT	CC	19.2
S444	Case	CT	TC	
S445	Case	CT	TC	22.1
S446	Case	CC	CC	38.4
S447	Case	CC	CC	12.6
S448	Case	CT	TC	35.5
S449	Case	CT	TC	40.6
S450	Case	CT	TC	47.7
S451	Case	TT	CC	14.9
S452	Case	CC	TC	25
S453	Case	TT	TC	35.1

S454	Case	CC	TC	38.4
S455	Case	CT	TC	20.4
S456	Case	CT	TC	52
S457	Case	CC	TC	32.8
S458	Case	CT	CC	26.3
S459	Case	TT	CC	20.2
S460	Case	CT	CC	
S461	Case	CC	TC	35
S462	Case	TT	TC	35
S463	Case	CT	TC	28.2
S464	Case	CT	CC	15.2
S465	Case	CC	TC	12.4
S466	Case	CC	TC	15.8
S467	Case	CT	TC	13.7
S468	Case	CT	TC	20
S469	Case	TT	TT	10.5
S470	Case	TT	TC	32.6
S471	Case	CC	TC	39.8
S472	Case	CT	TC	16.6
S473	Case	CT	CC	18.6
S474	Case	TT	TC	66.4
S475	Case	CT	TC	26.3
S476	Case	TT	TC	27.7
S477	Case	CT	TC	50.5
S478	Case	TT	TC	17
S479	Case	CC	CC	10.4
S480	Case	CT	CC	21.2
S481	Case	CT	CC	34.2
S482	Case	CT	TC	
S483	Case	CT	TC	12.6
S484	Case	CT	CC	35.4
S485	Case	TT	TC	28.3

S486	Case	TT	TC	13.5
S487	Case	TT	CC	45.5
S488	Case	TT	TC	37.4
S489	Case	TT	TC	42.8
S490	Case	CT	TT	40.2
S491	Case	CC	CC	40.3
S492	Case	TT	TC	26.8
S493	Case	TT	TC	41.2
S494	Case	CC	CC	35.6
S495	Case	CT	CC	36.7
S496	Case	CC	TC	14.8
S497	Case	TT	TC	38.9
S498	Case	TT	TC	16.9
S499	Case	CT	TC	26.6
S500	Case	CT	TT	10.9
S501	Case	CC	TC	20.2
S502	Case	CT	TC	40.8
