# Supplementary material for: Genetic association and computational analysis of CYP2R1 gene polymorphisms rs2060793 and rs12794714 with vitamin D deficiency and acute myocardial infarction in the Bangladeshi population: A case control study
Source: PLoS One. 2026 Jun 5;21(6):e0350994. doi: 10.1371/journal.pone.0350994 (PMC13240929; doi:10.1371/journal.pone.0350994)
Supplement: S4 Table — (PDF) [file pone.0350994.s005.pdf]

S4 Table: Combined effect of both SNPs on AMI risk

Genotype (r12794714)	Genotype (rs2060793)	Odds Ratio	95% CI	p-value
**CC**	**TT**	1(Ref.)		
**CC**	**CC**	1.47	0.53–4.24	0.465
**CC**	**TC**	1.37	0.52–3.72	0.531
**CT**	**CC**	2.05	0.81–5.49	0.139
**CT**	**TC**	2.03	0.83–5.19	0.126
**CT**	**TT**	0.46	0.13–1.60	0.228
**TT**	**CC**	2.22	0.82–6.30	0.121
**TT**	**TC**	1.54	0.56–4.42	0.411
**TT**	**TT**	0.6	0.073 – 3.55	0.586

$p<0.05$ was considered as level of significance.
