# Supplementary material for: Genetic association and computational analysis of CYP2R1 gene polymorphisms rs2060793 and rs12794714 with vitamin D deficiency and acute myocardial infarction in the Bangladeshi population: A case control study
Source: PLoS One. 2026 Jun 5;21(6):e0350994. doi: 10.1371/journal.pone.0350994 (PMC13240929; doi:10.1371/journal.pone.0350994)
Supplement: S5 Table — (PDF) [file pone.0350994.s006.pdf]

S5 Table: Prediction of transcription factor binding sites encompassing the variant position (rs2060793) using JASPAR

Name of Transcription Factor	Score	Relative Score	Predicted Sequence in ±50bp of targeted variant position
**SOX10**	6.091762	0.8244123	AACTCAAAGAT
**MZF1**	5.8375854	0.85181797	TTGGGA
**REL**	7.3118525	0.8267241	TGGGATTATC
**NFKB1**	3.6601326	0.8023697	TGGGATTATCC
**RBPJ**	4.4772024	0.83500266	TGGGAT
**NR2C2**	2.7233052	0.8695478	AGTTGG
**DUX4**	1.3257738	0.82012993	TAATCCCAACT
**NFIC**	4.80088	0.8359971	TTGGGA
**CRX**	11.739408	0.9734343	TGGGATTATC
**HNF4A**	10.136955	0.8698033	CTGATCTTTGAGTTG
